# Oncogene inactivation-induced senescence facilitates tumor relapse

**DOI:** 10.1038/s41467-026-75021-9

**Published:** 2026-07-15

**Authors:** Philipp Schmitt, Katrin Hönig, Maria Teresa Norcia, Marta F. Nogueira, Viktoria Flore, Inés Simó Vesperinas, Maria Villoro-Agud, Lushan Peng, Zuhal Safyürek, Mehreen Tariq, Ana Milojkovic, Kathleen Anders, Evelin Schröck, Sascha Sauer, Bora Uyar, Altuna Akalin, Gerald Willimsky, Simon Haas, Inmaculada Martínez-Reyes, Thomas Blankenstein

**Affiliations:** 1 Max-Delbrück Center for Molecular Medicine in the Helmholtz Association, Berlin, Germany; 2 Institute of Tumor Immunology, Charité – Universitätsmedizin Berlin, Campus Buch, Berlin, Germany; 3 Berlin Institute of Health (BIH) at Charité Universitätsmedizin Berlin, Berlin, Germany; 4 German Cancer Research Center (DKFZ), Heidelberg, Germany; 5 German Cancer Consortium (DKTK), partner site Berlin, a partnership between DKFZ and Charité-Universitätsmedizin Berlin, Berlin, Germany; 6 Department of Hematology, Oncology and Tumor Immunology, Charité – Universitätsmedizin Berlin, Berlin, Germany; 7 Berlin Institute for Medical Systems Biology, Max Delbrück Center for Molecular Medicine in the Helmholtz Association, Berlin, Germany; 8 Precision Healthcare University Research Institute, Queen Mary University of London, London, UK; 9 Institute for Clinical Genetics, University Hospital Carl Gustav Carus at TUD Dresden University of Technology and Faculty of Medicine of TUD Dresden University of Technology, Dresden, Germany; 10 ERN GENTURIS, Hereditary Cancer Syndrome Center Dresden, Dresden, Germany; 11 National Center for Tumor Diseases (NCT), NCT/UCC Dresden, a partnership between DKFZ, Faculty of Medicine and University Hospital Carl Gustav Carus, TUD Dresden University of Technology, and Helmholtz-Zentrum Dresden-Rossendorf (HZDR), Dresden, Germany; 12 German Cancer Consortium (DKTK), partner site: Dresden, and German Cancer Research Center (DKFZ), Heidelberg, Germany; 13 Cluster of Excellence ImmunoPreCept, Charité - Universitätsmedizin Berlin, Berlin, Germany; 14 Present Address: Translational Microbiome Research, Internal Medicine I and M3 Research Center, University Hospital Tuebingen, Tübingen, Germany; 15 Present Address: Department of Pediatric Oncology, Charité – Universitätsmedizin Berlin, Berlin, Germany

**Keywords:** Cancer metabolism, Cancer microenvironment, Senescence, Cancer

## Abstract

Oncogene-directed therapies can induce profound tumor regression in oncogene-addicted cancers, but their long-term benefit is often limited by resistance and relapse. Here we show that oncogene inactivation rapidly induces senescence and a pro-inflammatory senescence-associated secretory phenotype (SASP). In vivo, oncogene inactivation-induced senescence (OIIS) predisposes tumors to relapse, accompanied by polyploidy, chromosomal instability, acquisition of alternative oncogenic pathways including mouse double minute 2 homolog (Mdm2) upregulation, and tumor microenvironmental remodeling toward neovascularization and immunosuppression. Spectral flow cytometry reveals a shift from an immune-activated to an immunosuppressive milieu during relapse. OIIS features are also observed in human BRAF^V600E^ melanoma cells treated with vemurafenib, supporting clinical relevance. Together, our findings establish OIIS as a double-edged process: it initially restrains tumor growth but simultaneously creates conditions that favor recurrence. By defining the genetic, metabolic and microenvironmental hallmarks of OIIS, our study highlights adaptations to oncogene deprivation that limit the durability of targeted therapies.

## Abstract (web summary)

Oncogene targeted treatments can push cancer cells into a senescent, inflammatory state that initially halts tumor growth. Here, the authors show that this senescence program also creates conditions that enable tumor cells to escape, acquire new oncogenic traits, and ultimately drive relapse through genetic, metabolic and microenvironmental reprogramming.

## Introduction

Oncogene-directed therapy has emerged as a promising approach in cancer drug development^1–4^. However, despite its potential, clinical efficacy is often limited by resistance and relapse^5–8^. Targeted therapies can induce profound tumor regression in oncogene-addicted cancers, but residual disease often persists, eventually giving rise to recurrence. The mechanisms that enable cancer cells to adapt to oncogene inactivation, survive in a dormant state, and resume proliferation remain incompletely understood. In this study, we investigated these processes using a mouse cancer model in which cancer cell proliferation is driven by the oncogene SV40 large T antigen (Tag) in an inducible manner, allowing us to activate and inactivate the oncogene in vitro and in vivo. Importantly, this system mimics clinical scenarios where tumor growth is dependent on oncogene signaling and recapitulates oncogene addiction, a phenomenon well-documented in human cancers such as BRAF-mutant melanoma and KRAS-driven pancreatic cancer. Our results show that oncogene inactivation leads to the acquisition of cellular senescence and induction of a pro-inflammatory senescence-associated secretory phenotype (SASP). Senescence is widely defined as a durable form of growth arrest and is linked to extensive cellular stress that can be induced by different types of insults, including oncogenic activation, radiation, or chemotherapy^9^. Since the seminal work of Hayflick and Moorhead in 1961, our understanding of senescence has expanded, with increasing evidence for both beneficial and detrimental roles in aging and disease^10–12^.

In the cancer context, senescence plays a complex, dual role. Oncogene-induced senescence (OIS) is an early barrier to malignant transformation, enforcing cell-cycle arrest and, in some models, initiating immune-mediated clearance through SASP-driven recruitment of effector cells^13–15^. However, if senescent cells persist, their SASP can paradoxically remodel the tumor microenvironment, promoting inflammation, extracellular matrix degradation, and the secretion of growth-promoting factors that facilitate tumor progression^16–18^. Therapy-induced senescence (TIS) has historically been viewed as a favorable outcome of cancer treatment, suppressing tumor growth by arresting the proliferation of genetically unstable cancer cells^19^. However, mounting evidence suggests that TIS may instead promote long-term tumor progression. Cancer cells that survive therapy often acquire senescent features and may later escape this state, leading to more aggressive relapses^20–22^. In acute myeloid leukemia, chemotherapy induces a transient senescence-like state from which cells re-enter the cell cycle with enhanced stemness and resilience^22^. Similarly, in B-cell lymphoma, TIS leads to epigenetic reprogramming toward stemness, increasing tumor-initiating capacity^21^. Over time, senescent tumor cells can accumulate epigenetic “scars” that predispose them to senescence escape, chromatin remodeling, and oncogenic reactivation^23^. Beyond classical inducers of senescence, a growing body of work indicates that targeted oncogene inactivation itself can trigger senescence in oncogene-addicted tumors. This phenomenon has been described following c-MYC inactivation in lymphoma^24^, after BRAF inhibition in melanoma^25^ and after KRAS inhibition in pancreatic cancer^26^. Whether OIIS enforces durable growth arrest or instead creates a state prone to relapse has remained unclear.

Here, we address this gap using a controllable oncogene switch model in which SV40 large T antigen (Tag) drives tumor cell proliferation in an inducible manner. This system recapitulates oncogene addiction and allows us to study the effects of oncogene activation and inactivation in vitro and in vivo. Using epithelial (clone 4 gastric carcinoma) and mesenchymal (TTC#3055 spindle cell sarcoma) tumor cell lines, we demonstrate that OIIS predisposes tumors to relapse and is associated with adverse outcomes. We further explore how OIIS shapes tumor evolution, including chromosomal instability, acquisition of alternative oncogenic pathways, and metabolic adaptations. Finally, we validate key features of OIIS in human BRAF^V600E^ melanoma cells, confirming that our findings are relevant to oncogene-driven cancers.

## Results

### Oncogene inactivation induces senescence and SASP

**Fig. 1:**
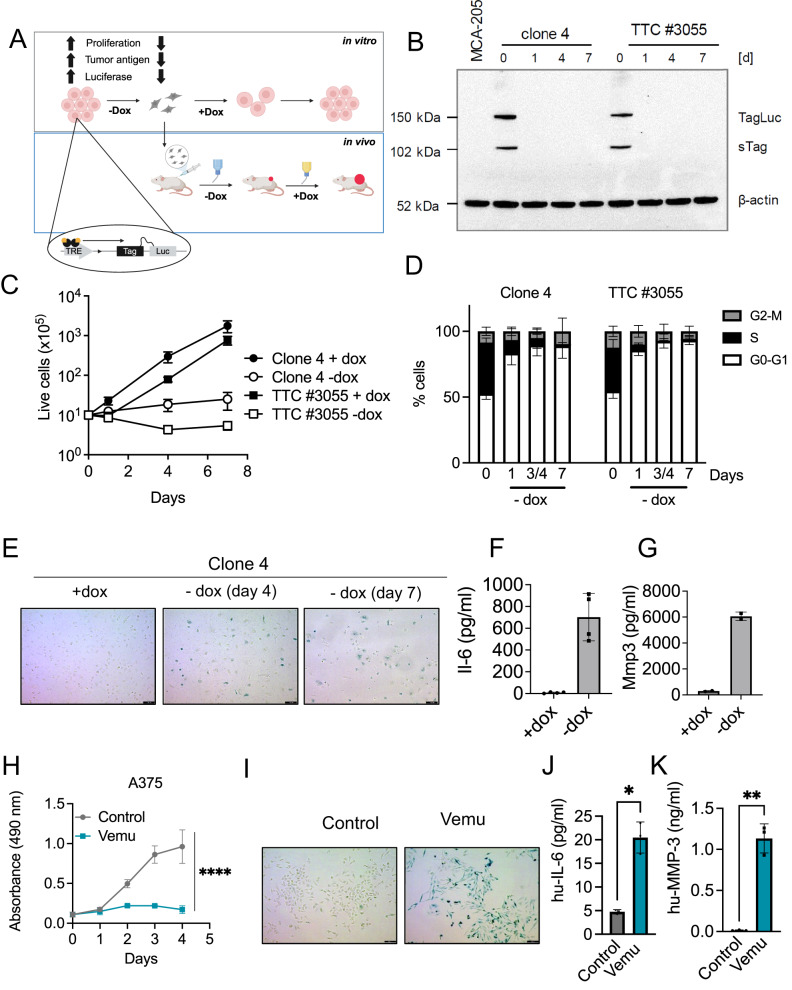
Oncogene inactivation induces senescence and a pro-inflammatory SASP. **A** Schematic of the mouse model. The model features the SV40 large T antigen (Tag) oncogene regulated by a tetracycline response element (TRE). Addition or withdrawal of doxycycline (dox) activates or inactivates the oncogene, respectively. Tag is fused to firefly luciferase (Luc), creating the TagLuc fusion protein, which serves as both a cancer driver and a reporter for intravital imaging. Oncogene activation and inactivation are reversible both in vitro and in vivo. Created in BioRender. Martínez-Reyes, I. (https://BioRender.com/9mldwgj) **B** Western blot analysis of TagLuc in clone 4 and TTC#3055 cells cultured in the presence of dox or at different time points after dox withdrawal. β-actin was used as a loading control. MCA-205 served as negative control. A shorter splicing product of Tag ( ~ 100 kDa), sTagLuc, was detected. Shown is one representative experiment out of four independent experiments. **C** Clone 4 and TTC#3055 cells were grown in the presence or absence of dox and live cell number was assessed at different time points. *n* = 3 biological replicates, shown is mean ± s.e.m. **D** Cell-cycle phase distribution was determined by BrdU incorporation following TagLuc inactivation. Error bars indicate mean ± standard deviation, *n* = 3 independent experiments. **E** SA-β-galactosidase staining of clone 4 cells grown with dox or after four or seven days post-TagLuc inactivation. Scale bar, 100 μm. **F**, **G** Clone 4 cells were cultured with or without dox for two weeks. Conditioned medium was generated and normalized to cell number (2 × 10^5^ cells/ml). Il-6 (F) and Mmp3 (G) secretion was analyzed via ELISA. Error bars indicate mean ± standard deviation. Il-6: *n* = 4, pooled data from two independent experiments comprising two replicates. Mmp3: *n* = 2 technical replicates from one experiment. **H**, Proliferation of A375 melanoma cells treated with vemurafenib (Vemu, 1 µM) or vehicle control was measured by MTS assay at the indicated time points (mean ± s.d.; *n* = 3 independent experiments, 2way ANOVA with multiple comparisons). ****p < 0.0001. **I**, Bright-field microscopy of A375 cells treated with Vemu showing flattened morphology, enlarged cell shape and positive SA-β-galactosidase staining. Scale bar, 100 μm **J**, **K** Secreted SASP factors hu-IL-6 (J, *p *= 0,014) and hu-MMP-3 (K, *p *= 0,0088) measured by ELISA in the supernatant of control vs. Vemu-treated cells for 15 days (mean ± s.d.; *n* = 3 independent experiments, two-tailed t-test was used). Source data for this figure are provided as a Source Data file.

To study the mechanisms enabling cancer cells to adapt to oncogene inactivation, we used cancer cells isolated from tumors that grew sporadically in transgenic mice^27,28^. Specifically, we used cells from a gastric carcinoma (clone 4) and from a spindle cell sarcoma isolated from the snout (TTC#3055). These established tumor cell lines express the oncogene Tag fused to the reporter gene firefly luciferase (TagLuc) under the tetracycline response element (TRE) together with the rtTA2S-M2 transactivator (rtTA). In clone 4 cells, the loxP-flanked stop cassette was excised by transient gene transfer in cultured cells with a Cre recombinase-encoding adenovirus. This system allows conditional activation or inactivation of the oncogene by adding or withdrawing doxycycline (dox), respectively, both in vitro and in vivo. Fusion of Tag to luciferase enables monitoring of tumor cell growth in vivo by bioluminescence imaging upon subcutaneous transplantation (Fig. 1A). This model provides an ideal setting to analyze the effects of oncogene inactivation on cancer cells and tumor development. We first confirmed loss of TagLuc expression, indicating oncogene inactivation, as early as one day after dox withdrawal in clone 4 and in TTC#3055 cells (Fig. 1B and Supplementary Fig. 1A). Following oncogene inactivation, cellular proliferation was halted, and cells progressively exhibited cell cycle arrest (Fig. 1C, D, and Supplementary Fig. 1B). Cells became enlarged, flattened, and displayed an irregular morphology, characteristic of a senescent phenotype. Following TagLuc inactivation, a significant increase in senescence-associated β-galactosidase (SA-β-gal) activity was detected in clone 4 cells (Fig. 1E and Supplementary Fig. 1C, D).

To further validate senescence induction, we analyzed the expression of Cdkn1a (p21) and Cdkn2a (p16), two key cell cycle regulators associated with senescence^29^. Consistent with senescence acquisition, we observed increased p21 protein levels after oncogene inactivation (Supplementary Fig. 1E). However, p16 protein was downregulated, a finding that deviates from the canonical senescence signature (Supplementary Fig. 1E). Analysis of mRNA levels confirmed an upregulation of p21 and a downregulation of p16 following TagLuc inactivation (Supplementary Fig. 1F). A hallmark of senescent cells is the SASP, characterized by cytokine and matrix-remodeling enzyme secretion^17^. We analyzed the SASP by generating conditioned medium from proliferating (TagLuc-expressing) and senescent (TagLuc-negative) clone 4 cells. Using a semi-quantitative cytokine array, we observed an increase in the secretion of interleukin-6 (Il-6), matrix metalloprotease 2 (Mmp2), and matrix metalloprotease 3 (Mmp3) in senescent cells (Supplementary Fig. 1G). ELISAs confirmed elevated Il-6 and Mmp3 secretion after senescence induction (Fig. 1F, G).

To confirm the relevance of our observations in a human context, we next assessed whether therapeutic oncogene inhibition induces senescence and SASP in human melanoma cells. Treatment of A375 cells with the mutant BRAF inhibitor vemurafenib led to a strong reduction in proliferation compared to vehicle-treated controls (Fig. 1H). Vemurafenib-treated cells acquired classical features of senescence, including SA-β-gal staining and a flattened, enlarged morphology (Fig. 1I, and Supplementary Fig. 1H). Immunoblotting revealed a progressive decrease in phosphorylated Rb (pRb) without upregulation of p21 or p16 (Supplementary Fig. 1I, J), consistent with the acquisition of a non-canonical senescence program reported in melanoma cells upon mutant BRAF inhibitors treatment^30^. To evaluate SASP induction in human cells, we measured the secretion of IL-6 and MMP-3 in A375 cells treated with vemurafenib. Both factors were upregulated upon treatment, confirming the acquisition of a SASP in this model (Fig. 1J, K).

Collectively, the concordant findings in mouse and human cells show that oncogene inactivation leads to the acquisition of a senescent phenotype.

### TagLuc inactivation-mediated senescence promotes major transcriptomic changes and the acquisition of a plurimetabolic state

**Fig. 2:**
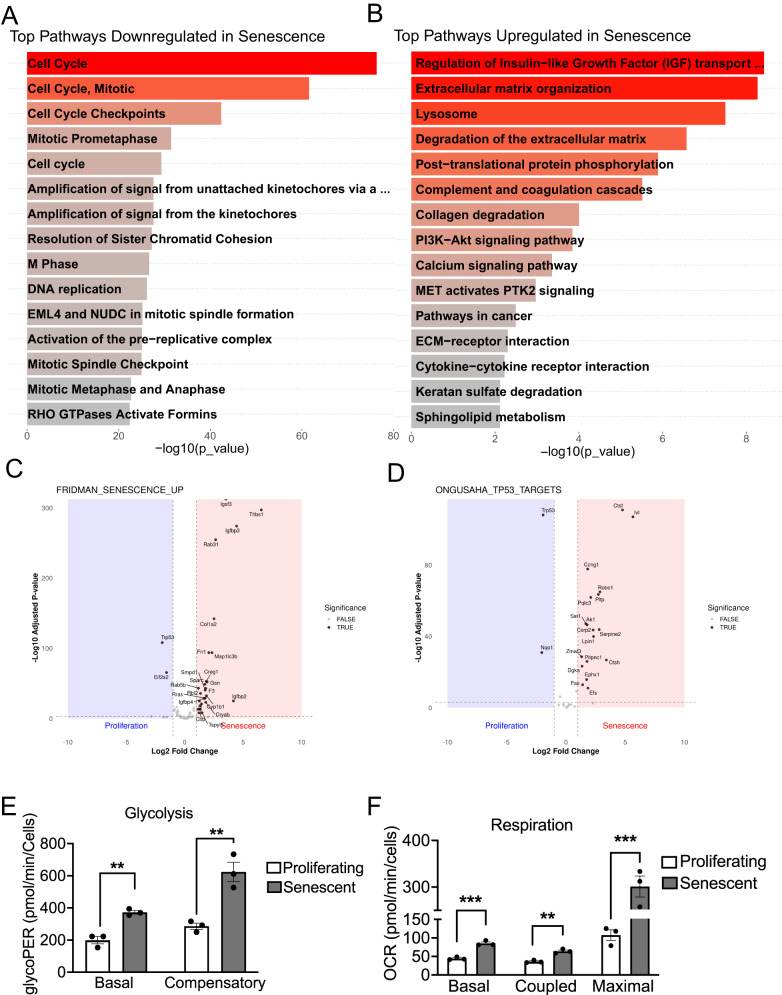
Senescence is accompanied by major transcriptional changes and the acquisition of a plurimetabolic state. **A**, **B** Enriched pathway terms (KEGG/Reactome) among the downregulated (A) and upregulated (B) genes in senescent (TagLuc-negative, -dox) cells compared to proliferating (TagLuc-expressing, +dox) cells. For clone 4 cells, RNA-seq was performed on three independently processed replicate cultures. A complete dataset of differentially expressed genes can be found in Supplementary Data 1. **C**, **D** Gene-set enrichment analysis using the FRIDMAN_SENESCENCE_UP and ONGUASAHA_TP53_TARGETS gene sets in senescent (TagLuc-negative, –dox) versus proliferating (TagLuc-expressing, +dox) clone 4 cells. *n* = 3 biological replicates. **E** Basal (*p* = 0,0025) and compensatory (*p* = 0,0058) glycolysis in senescent (TagLuc-negative, -dox) cells compared to proliferating (TagLuc expressing, +dox) cells. Data are mean ± s.e.m; *n* = 3 independent experiments, two-tailed t-test was used. **F** Basal (*p* = 0,00083), coupled (*p* = 0,0021), and maximal respiration (*p* = 0,0019) in senescent (TagLuc-negative, -dox) cells compared to proliferating (TagLuc-expressing, +dox) cells. Data are mean ± s.e.m; *n* = 3 independent experiments, two-tailed t-test was used. Source data are provided as a Source Data file for E and F. Details on data analysis and statistical evaluation for A-D are provided in the corresponding Methods section.

To further characterize the phenotype of OIIS, we performed RNA sequencing of clone 4 cells in two conditions: proliferating (TagLuc-expressing, +dox) and senescent (TagLuc-negative, -dox). Differential gene expression analysis revealed profound transcriptional reprogramming, with 1,067 genes significantly upregulated (p-adj <0.001, log2 fold-change > 2) and 928 genes significantly downregulated (p-adj <0.001, log2-fold-change < −2) in senescent cells compared to proliferating cells (Supplementary Fig. 2A; and Supplementary Data 1). Gene set enrichment analysis (GSEA) demonstrated that pathways related to cell cycle regulation, mitosis, and DNA replication were among the most significantly downregulated in senescent cells, consistent with the G0/G1 arrest observed after oncogene inactivation (Fig. 2A). Conversely, senescent cells showed marked upregulation of pathways involved in extracellular matrix (ECM) remodeling, including keratan sulfate degradation, ECM-receptor interaction, collagen degradation, and matrix organization (Fig. 2B). Additionally, senescent cells exhibited enrichment of inflammatory signaling and growth factor-related pathways such as cytokine-cytokine receptor interactions, complement activation, PI3K-AKT signaling, and insulin-like growth factor (IGF) transport (Fig. 2B). Sphingolipid metabolism, which has been previously implicated in the regulation of senescence, was also upregulated, reflecting changes in membrane dynamics and metabolic adaptation^31^. The SASP was confirmed by increased expression of *Mmp13*, *Mmp3*, *Igfbp2*, *Il6*, and other inflammatory mediators (Supplementary Fig. 2B).

Consistent with senescence induction, the FRIDMAN_SENESCENCE_UP gene set was significantly enriched in senescent cells (Fig. 2C)^32^. We next analyzed p53 pathway activity using the ONGUASAHA_TP53_TARGETS gene set, which revealed selective enrichment of senescence-associated p53 targets in senescent cells (Fig. 2D)^33^. Analysis of the Cancer Single-Cell State Atlas (CancerSEA) revealed that senescent cells downregulate cell cycle, DNA repair, DNA damage response, and proliferation signatures, while upregulating pathways linked to hypoxia, angiogenesis, invasion, and inflammation (Supplementary Fig. 2C, D)^34^.

In addition, we analyzed the metabolic phenotype of proliferating versus senescent clone 4 cells. Senescent cells displayed a higher basal glycolytic rate, as well as increased compensatory glycolysis after mitochondrial inhibition with rotenone and antimycin A (Fig. 2E; and Supplementary Fig. 2E). We also observed that basal, coupled, and maximal oxygen consumption rates were significantly higher in senescent cells compared to proliferating controls, indicating enhanced mitochondrial respiration (Fig. 2F; Supplementary Fig. 2F). To functionally test the dependency of senescent cells on glycolysis and respiration, we treated cells with 2-deoxyglucose (2-DG) or rotenone/antimycin A (R/A) individually. Senescent cells displayed increased sensitivity to both glycolysis inhibition and mitochondrial respiration blockade compared to proliferating cells, with a significant increase in cell death upon either treatment (Supplementary Fig. 2G). This finding supports the dual activation of glycolysis and oxidative phosphorylation, confirming that senescent cells acquire a plurimetabolic state for survival.

### Oncogene inactivation-induced senescence predisposes to cancer relapse

**Fig. 3:**
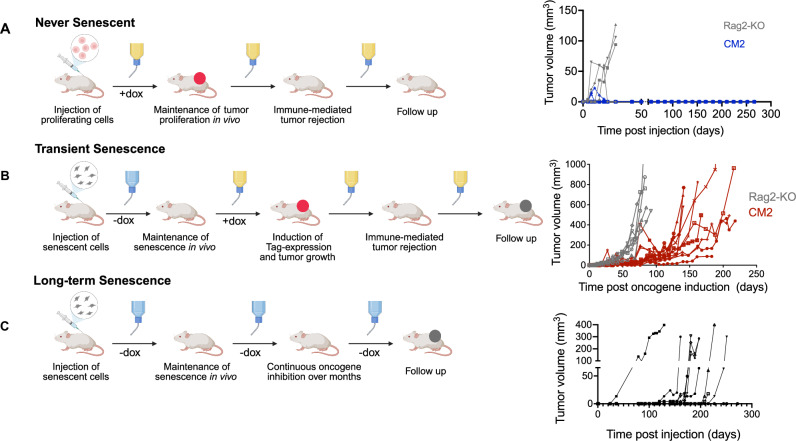
Distinct senescence regimens shape tumor regression and relapse in vivo. **A** *Never-senescent condition*. Schematic (left) and tumor growth curves (right) for mice injected s.c. with proliferating clone 4 cells (TagLuc-expressing, +dox) and maintained on doxycycline throughout. Continuous +dox preserves TagLuc expression and tumor cell proliferation in both CM2 and Rag2-KO mice. In immunocompetent CM2 mice (blue, *n* = 8 mice), tumors initially grow but are subsequently eliminated by Tag/SV40-specific immune responses, whereas in Rag2-KO mice (gray, *n* = 4 mice) tumors grow progressively. No tumor relapse is observed in CM2 mice during long-term follow-up. **B** *Transient OIIS condition*. Schematic (left) and tumor growth curves (right) for mice injected with senescent clone 4 cells generated by 14 days of dox withdrawal in vitro (dox off). After injection, mice are kept off dox for four weeks to maintain OIIS in vivo, and then switched to +dox to reactivate TagLuc and induce tumor growth. In CM2 mice (red, *n* = 13 mice), tumors initially expand under +dox and are then rejected by the immune system, but after a prolonged latency tumors relapse. In Rag2-KO mice (gray, n = 6 mice), tumors grow continuously without regression, consistent with the absence of adaptive immunity. Two mice from the CM2 group were excluded from the analysis because they died from causes unrelated to tumor disease. **C** *Long-term OIIS condition*. Schematic (left) and tumor growth curves (right) for mice injected with senescent clone 4 cells and kept off dox for the entire duration of the experiment, resulting in sustained oncogene inactivation in vivo. Despite continuous TagLuc suppression, tumors eventually arise in most CM2 mice (black, 9 out of 12 mice) after a long latency, indicating senescence escape and oncogene-independent outgrowth. Source data for this figure are provided as a Source Data file. Created in BioRender. Martínez-Reyes, I. (https://BioRender.com/w37epud).

To analyze how OIIS influences tumor relapse, we established three complementary in vivo conditions (Fig. 3): a) a never-senescent setting with continuous oncogene expression ( + dox throughout); b) a transient OIIS setting in which TagLuc is switched off and later reactivated (dox off → on), and c) a long-term OIIS setting in which TagLuc remains suppressed for the entire experiment (dox off throughout). These three conditions allow us to compare tumors that never experience senescence, tumors that undergo a reversible senescent phase, and tumors under a long-term senescent state.

First, we injected proliferating clone 4 cells (TagLuc expressing, +dox) into CM2 and Rag2-KO mice, while maintaining dox throughout (Fig. 3A). CM2 mice ubiquitously express the rtTA transactivator and are therefore tolerant to it, eliminating the possibility of immune responses against the transactivator itself. Tumor burden was quantified by tumor volume and TagLuc-derived bioluminescent flux, the latter directly reporting the in vivo burden of oncogene-expressing tumor cells, as the firefly luciferase is fused to the oncogene Tag. In CM2 mice, tumors initially grew but were consistently rejected by immune responses targeting TagLuc, shown by staining of peptide IV-MHC I tetramer^+^ CD8⁺ T-cells (Fig. 3A and Supplementary Fig. 3A, B). Importantly, no tumor relapse was observed in these mice over a long observation period. In Rag2-KO mice, tumors grew progressively consistent with the absence of immune-mediated clearance (Fig. 3A, and Supplementary Fig. 3E).

Next, we withdrew dox from cultured clone 4 cells for 14 days to induce senescence in vitro (Fig. 3B). Senescent cells were then injected into immunocompetent CM2 mice and immunodeficient Rag2-KO mice. After injection, the cells were kept in a senescent state in vivo by maintaining mice off dox for four weeks. We then introduced dox via the drinking water to reactivate TagLuc expression and stimulate tumor growth. This design therefore models a transient phase of OIIS, in which senescence is imposed during dox withdrawal (in vitro and in vivo) and is reversed upon reactivation of TagLuc once dox is introduced in vivo. In CM2 mice, tumors initially grew after dox reintroduction but subsequently regressed due to immune-mediated rejection, as confirmed by bioluminescence imaging and measurement of tumor volume (Fig. 3B and Supplementary Fig. 3C). Re-introduction of dox restored TagLuc expression, rendering tumor cells immunogenic and leading to their elimination by Tag/SV40-specific CD8⁺ T cells (Supplementary Fig. 3D). In Rag2-KO mice, by contrast, tumors grew progressively without regression (Fig. 3B, and Supplementary Fig. 3F). Notably, after a prolonged latency, tumor relapse occurred in all CM2 mice injected with transiently senescent clone 4 cells. Similar results were obtained with TTC#3055 cells: after oncogene reactivation in vivo, tumors in CM2 mice initially grew, regressed, and later relapsed, whereas in Rag2-KO mice, tumors grew continuously (Supplementary Fig. 3G, H).

To summarize, continuous oncogene expression in the absence of any senescent phase leads to durable immune-mediated eradication (in CM2 mice) but not to relapse. A preceding transient phase of OIIS, on the other hand, leads to tumor relapse after initial regression. These findings indicate that it is specifically a preceding transient OIIS phase that creates a unique condition which predisposes tumors to late relapse despite initial immune clearance.

We next tested whether long-term senescence alone could lead to tumor formation without oncogene reactivation (Fig. 3C). In contrast to the transient OIIS protocol above, where dox withdrawal and re-addition impose a reversible senescent phase, here oncogene expression remains suppressed throughout the entire experiment. Clone 4 cells maintained off dox for 14 days were injected into CM2 mice, which were then kept continuously off dox. Remarkably, tumors developed in 9 out of 12 mice after a long latency, despite continuous oncogene suppression. This indicates that a subset of OIIS cells can eventually undergo senescence escape, i.e., lose their growth arrest and re-enter the cell cycle in vivo, even without reactivation of the original oncogene. To explore the underlying mechanism, we re-isolated cell lines from relapsed tumors in both experimental conditions (transient and long-term senescence). In four of five cell lines, TagLuc expression was undetectable by luciferase activity and mRNA analysis (Supplementary Fig. 3I, J), consistent with the absence of bioluminescence in several relapsed tumors. Thus, tumor outgrowth after OIIS can occur independently of the original oncogene, suggesting selection for alternative oncogenic pathways. Together, these results demonstrate that OIIS creates a cellular state that fosters oncogene bypass and tumor relapse.

To determine whether vemurafenib-induced OIIS is transient, we first treated A375 melanoma cells with vemurafenib in vitro to induce growth arrest and acquisition of senescence markers. Upon withdrawal of vemurafenib, cells resumed proliferation (Supplementary Fig. 3K), indicating that the senescent state induced by mutant BRAF inhibition is reversible. In a complementary in vivo experiment, we pretreated A375 cells with vemurafenib in vitro to induce senescence, implanted them subcutaneously, and continued vemurafenib treatment of recipient mice for four weeks (Supplementary Fig. 3L). As shown in Supplementary Fig. 3M, tumors formed and grew despite this extended senescence-inducing regimen, further supporting the transient nature of therapeutic OIIS in this model. These findings demonstrate that BRAF inhibitor–induced OIIS in human melanoma cells is not durably tumor-suppressive but instead creates a state that permits senescence escape and tumor regrowth.

### Cancer cells from relapsed tumors depend on Mdm2 and display polyploidy

**Fig. 4:**
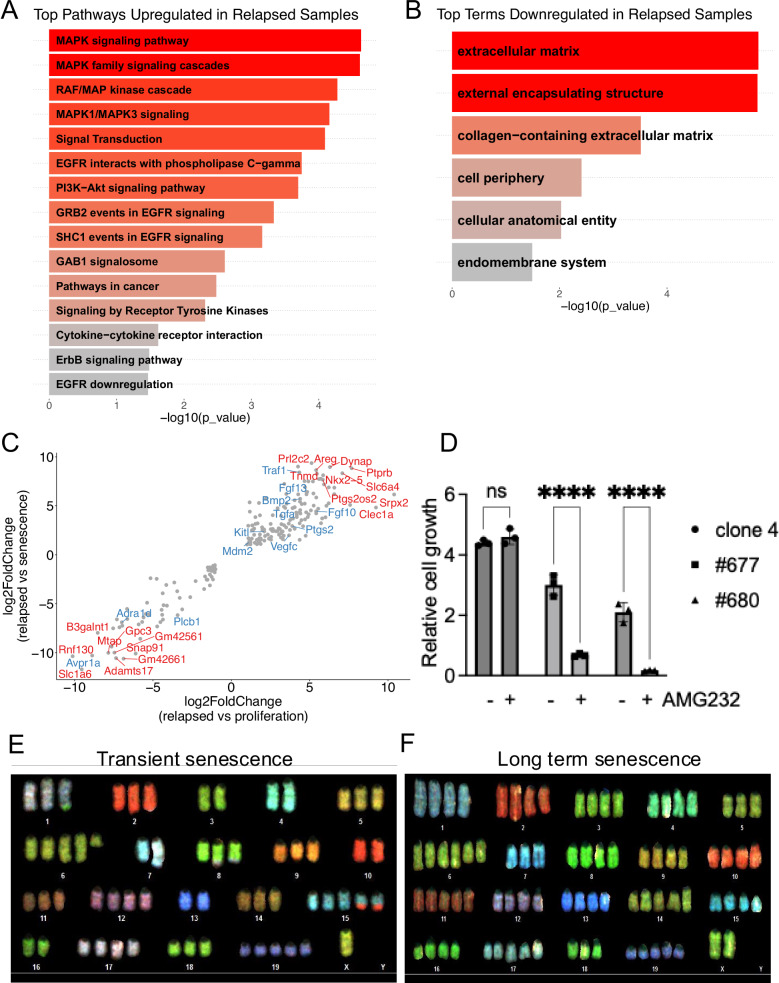
Cancer cells re-isolated from relapsed tumors depend on Mdm2 and acquired polyploidy. **A**, **B** Top enriched pathways among genes upregulated (**A**) or downregulated (**B**) in relapse-derived cancer cell lines compared to both parental proliferating (TagLuc-expressing, +dox) and senescent (TagLuc-negative, –dox) clone 4 cells. RNA-seq was performed on three independently processed replicate cultures. Pathway enrichment was based on significantly differentially expressed genes (adjusted *p* < 0.05, |log₂FC | > 1). **C** Genes that are upregulated and downregulated in relapsed cancer cell lines compared to proliferating (TagLuc-expressing, +dox) and senescent (TagLuc-negative, -dox) clone 4 cells, *n* = 3 biological replicates. Genes in red represent the top 10 most strongly up- or downregulated genes across both comparisons; genes in blue are additional relevant candidates preselected as potential therapeutic targets. **D** Relative cell growth of parental clone 4 cells and cancer cell lines from relapsed tumors (#677, #680) following treatment with the Mdm2 inhibitor AMG-232 (1 µM for 72 h). Cell growth was calculated by comparing the cell number after treatment to the initial cell number. Data are shown as mean ± SD (*n* = 3 independent experiments, 2way ANOVA with multiple comparisons). ****p < 0.0001. **E**, **F** Representative spectral karyotyping (SKY) images of relapse-derived cell lines from tumors that emerged after transient TagLuc inactivation (**E**) or long-term TagLuc inactivation (**F**). Images were obtained from 8–10 randomly selected metaphase spreads per cell line, with one representative example shown. Supplementary Data 4 details the chromosomal aberrations observed in the 10 selected metaphase chromosomes of the parental Clone 4 cells. Source data for this figure are provided as a Source Data file. Details on data analysis and statistical evaluation for A-C are provided in the corresponding Methods section.

To investigate the molecular mechanisms supporting tumor relapse after OIIS, we analyzed transcriptional and cytogenetic features of cancer cell lines derived from relapsed tumors. We compared these lines to the parental proliferating clone 4 cells cultured in the presence of dox and to senescent clone 4 cells maintained off dox. Transcriptomic profiling revealed 143 genes significantly upregulated and 67 genes downregulated in relapsed cells relative to both parental and senescent populations (Supplementary Data 2). Pathway enrichment analysis of these differentially expressed genes indicated that relapsed tumors acquired molecular signatures associated with enhanced proliferation and growth factor signaling. Among the top upregulated pathways were MAPK signaling, RAF/MAPK kinase cascades, PI3K-AKT signaling and cytokine-receptor interactions (Fig. 4A). This profile reflects the activation of pro-growth signaling networks that are typically suppressed during senescence. Conversely, genes related to extracellular matrix organization, collagen-containing structures, and cellular adhesion were significantly downregulated in relapsed tumors (Fig. 4B). This shift suggests that relapsed tumors not only reactivate proliferative programs but also remodel their microenvironment, potentially facilitating tumor growth. Of note, gene set enrichment analysis comparing relapsed samples to the senescent state showed that relapsed tumors still show enrichment of senescence-related genes among the genes upregulated (Supplementary Fig. 4A). These findings suggest that relapsed cells acquire a hybrid state, maintaining residual senescence features, while engaging proliferation and growth signaling programs. We next assessed the metabolic changes in relapsed tumors, which showed a marked shift in metabolic pathway usage (Supplementary Fig. 4B). While glycosaminoglycan metabolism, sphingolipid metabolism, and keratan sulfate metabolism were downregulated, relapsed cells upregulated nucleotide metabolism, amino acid metabolism, and folate cycle pathways, reflecting increased anabolic activity to support proliferation (Supplementary Fig. 4B).

Among the upregulated genes in relapsed tumors, mouse double minute 2 homolog (Mdm2) emerged as a potential oncogenic driver (Fig. 4C). Since Mdm2 is a key negative regulator of p53, its increased expression could compensate for the loss of Tag-mediated p53 suppression following oncogene inactivation. To test this hypothesis, we treated relapsed tumor-derived cell lines with the Mdm2 inhibitor AMG-232^35^. The growth of relapsed cells was significantly impaired upon Mdm2 inhibition, whereas the parental clone 4 cells remained unaffected (Fig. 4D). These results confirm that relapsed tumors acquire an Mdm2-dependent proliferative advantage.

To evaluate whether genomic instability contributes to relapse, we performed multicolor karyotyping on the tumor-derived cell lines. Parental clone 4 cells maintained under continuous oncogene expression exhibited a near-diploid karyotype (Supplementary Fig. 4C). In contrast and in agreement with previous findings, relapsed tumor-derived cells displayed extensive chromosomal abnormalities and polyploidy (Fig. 4E, F and Supplementary Fig. 4D–F)^36,37^. Both transient- and long-term-senescence–derived relapsed cells exhibited dramatic chromosomal rearrangements and increased chromosome numbers, consistent with the acquisition of highly polyploid genomes. Despite the restoration of Rb and p53 function after Tag inactivation, these relapsed tumors retained complex structural aberrations and genomic instability. Altogether, these findings reveal that tumor relapse after OIIS is associated with a combination of genomic instability, metabolic reprogramming, and the activation of alternative oncogenic pathways such as Mdm2 upregulation.

### Distinct immune microenvironment landscapes characterize progressive tumors, remission and relapse

**Fig. 5:**
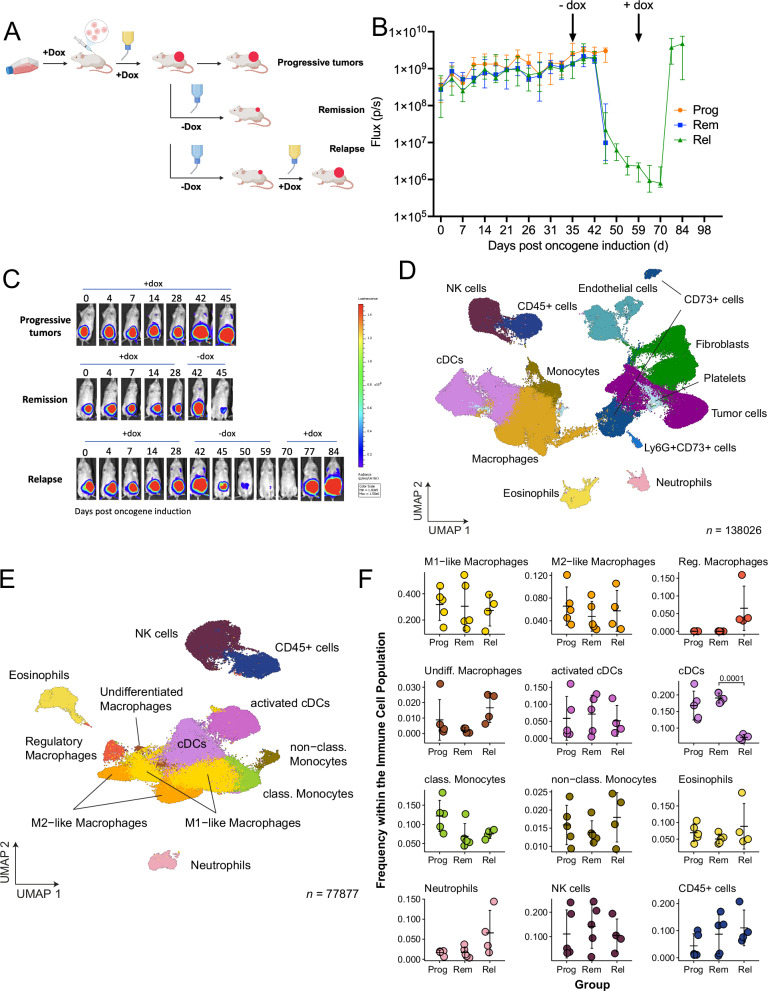
Immune infiltration in progressive tumors, remission and relapse. **A** Schematic representation of the OIIS mouse cancer model and relapse design. Clone 4 cancer cells cultured in the presence of doxycycline (dox) were subcutaneously injected (1 × 10⁵ cells in Matrigel) into Rag2-KO mice. Dox (200 µg/ml) was provided in the drinking water at tumor inoculation. In the remission and relapse groups, dox was withdrawn once tumors reached a clinically relevant size to induce OIIS. In the relapse group, oncogene expression was re-induced by re-administering dox once minimal BL signal was detectable. Created in BioRender. Martínez-Reyes, I. (https://BioRender.com/nrmknvb) **B** BL kinetics of Rag2-KO mice in the progressive (Prog; *n* = 5 mice), remission (Rem; *n* = 5 mice) and relapse (Rel; *n* = 6 mice) groups. Dox was removed at day 42 from the remission and relapse groups and re-administered at day 70 in the relapse group. Tumor plugs were isolated at day 45 from the progressive and remission groups and at day 102 from the relapse group. Shown is median with 95% CI. **C** Representative BL images of Rag2-KO mice from each group (exposure time: 60 s). The day after initial oncogene induction is indicated above each mouse. **D** UMAP representation of the overall cellular landscape. Recorded cells were processed with PICtR; out of 1,024,994 cells, 138,026 sketched cells are displayed. **E** UMAP representation of immune cells. Out of 318,115 cells, 77,877 sketched cells are displayed. **F** Frequency of the immune cell types identified in panel E within each experimental group (*n* = 5 biological replicates per group). One sample from the relapse group was excluded from spectral flow cytometry analysis due to clogging. *P* values were determined with a two-sided Welch’s *t*-test and corrected according to Benjamini-Hochberg. Error bars indicate the mean and standard deviation. cDCs: conventional dendritic cells, NK cells natural killer cells, non-class./class. Monocytes: non-classical/classical monocytes, UMAP Uniform Manifold Approximation and Projection. Source data for this figure are provided as a Source Data file.

To investigate how the tumor microenvironment contributes to OIIS during remission and to the subsequent relapse, we analyzed the immune and stromal cell populations across three experimental groups of Rag2-KO tumor-bearing mice. In the first group, tumors were maintained in a proliferative state through continuous dox administration, allowing persistent oncogene expression and progressive tumor growth. In the second group, once tumors were established, dox was withdrawn to inactivate the oncogene and induce remission. In the third group, following a remission phase induced by dox withdrawal, oncogene expression was reactivated to mimic relapse, providing a model to study the microenvironment during tumor regrowth. A schematic of this experimental setup is shown in Fig. 5A. Longitudinal bioluminescence imaging and tumor measurements confirmed progressive tumor growth upon sustained oncogene expression; tumors in the remission group regressed following oncogene inactivation; and tumors in the relapse group re-emerged after oncogene reactivation (Fig. 5B–C and Supplementary Fig. 5A).

To characterize the tumor microenvironment across progression, remission and relapse, we performed spectral flow cytometry of extracted tumor plugs (tumor cells were always injected in matrigel). Uniform Manifold Approximation and Projection (UMAP) visualization revealed the global composition of the tumor microenvironment, including immune, stromal, and endothelial populations that were identified using lineage markers (Fig. 5D and Supplementary Fig. 5B). UMAPs stratified by condition highlighted shifts in the cellular composition of relapsed tumors (Supplementary Fig. 5C). Interestingly, we observed a trend towards reduced endothelial cells upon induction of remission, followed by a significant increase in relapsed tumors (Supplementary Fig. 5D), likely reflecting transient vascular disruption after extensive tumor cell death following oncogene inactivation and subsequent neovascularization during tumor regrowth. Focusing on the immune compartment, we identified diverse cell types including NK cells, conventional and activated dendritic cells (cDCs), M1-like and M2-like macrophages, regulatory and undifferentiated macrophages, eosinophils, neutrophils and classical and non-classical monocytes (Fig. 5E and Supplementary Fig. 5E). Relapsed tumors displayed the most notable changes in the relative abundance of these populations (Fig. 5F and Supplementary Fig. 5F). In particular, we observed a significant reduction in cDCs and an increase in regulatory macrophages in relapsed tumors, pointing to a shift towards a more immunosuppressive microenvironment (Fig. 5F). These findings indicate that tumor relapse after oncogene inactivation is not solely driven by cell-intrinsic resistance mechanisms but also shaped by dynamic remodeling of the immune microenvironment.

## Discussion

In this study, we analyzed the effects of oncogene inactivation in vitro and in vivo and found that acquisition of cellular senescence, together with a pro-inflammatory SASP and genetic and metabolic alterations, are key adaptive mechanisms. Our results align with a broader spectrum of work showing that suppression of frequent oncogenes such as c-MYC, BRAF, or KRAS induces senescence programs that contribute to tumor regression but can also promote therapy resistance^25,38–41^. In our models, oncogene inactivation induced hallmark features of senescence, including cell cycle arrest, enlarged morphology, and SA-β-gal activity. Yet, unlike canonical senescence, p16 was consistently downregulated. In our mouse model system, this is explained by the regulatory interplay between Tag, p53, and Rb. SV40 Tag binds and functionally inactivates both p53 and Rb^42–44^, thereby preventing stress-induced apoptosis and cell-cycle arrest^45^. Tag binding stabilizes p53 protein but abolishes its transcriptional activity. Upon Tag withdrawal, this inhibition is released, resulting in selective enrichment of p53 target gene expression despite reduced p53 levels, creating a p21-dependent, but p16-independent senescence state. The downregulation of p16 is consistent with a feedback loop whereby Rb negatively regulates p16 transcription. In Rb-deficient or Tag-expressing cells, p16 accumulates due to loss of repression; conversely, restoration of Rb function when Tag is switched off suppresses p16 expression^46,47^. Thus, our system reflects how viral oncogene withdrawal reshapes tumor suppressor regulation, shifting senescence away from canonical p53–p16 signaling. We found that a similar noncanonical senescence program operates in human melanoma cells. In A375 BRAF^V600E^ melanoma cells, p16 also failed to increase upon oncogene inactivation, consistent with previous findings and the frequent p16 loss in melanoma cell lines^30,48^. Instead, vemurafenib-induced senescence was mediated by cyclin D1/p-Rb inhibition without robust activation of the DNA damage/p53/p21 axis in A375 cells. Together, these findings highlight that senescence can proceed via alternative, noncanonical pathways depending on the oncogenic context, with implications for therapy resistance.

Our results show that OIIS has profound consequences for disease progression in vivo. OIIS predisposed to tumor relapse from previously regressed tumors, resulting in significantly worse prognosis. These findings extend growing evidence that senescence can paradoxically fuel relapse. Much of our current understanding stems from OIS, initially defined as a potent tumor-suppressive barrier halting premalignant cell proliferation^49,50^. However, accumulating evidence shows that senescent cells can exert pro-tumorigenic effects through SASP-mediated remodeling of the microenvironment and cell-intrinsic adaptations^51–54^. Senescence-associated reprogramming, for instance, promotes stemness and tumor-initiating capacity^21^. In line with this, chemotherapy-induced senescence in acute myeloid leukemia and B-cell lymphoma generates cells that can later re-enter the cell cycle with increased stemness and tumorigenicity^21,22^. Here, we demonstrate that OIIS similarly predisposes tumors to relapse, extending tumor-promoting features of senescence beyond OIS and TIS.

Several mechanisms likely contribute. Previous studies showed that chemotherapy-induced senescence often yields polyploidy, enabling re-entry into the cycle, whereas non-polyploid senescent cells remain arrested^37,55,56^. In our model, relapsed cells displayed extensive chromosomal amplification, a hallmark of advanced, therapy-resistant tumors^57^. Recent work showed that extrachromosomal DNA (ecDNA) generates oncogene dosage heterogeneity affecting adaptation to therapy in MYCN-amplified cancers^58^. Together, these findings highlight how genomic alterations such as polyploidy, chromosomal instability, and ecDNA levels provide routes for tumor cells to persist and adapt under therapeutic pressure. Acquired polyploidy likely facilitates bypass of senescence through activation of alternative oncogenic pathways. Among these, Mdm2 emerged as a key driver. Mdm2 antagonizes p53 and is frequently overexpressed in cancers^59^. Accordingly, pharmacological Mdm2 inhibition with AMG-232 selectively killed relapsed cells, while parental cells remained unaffected. This dependence reflects the fact that, in our model, TagLuc inactivation restores p53 activity, and relapsed cells must therefore re-establish functional p53 suppression to escape OIIS. This aligns with prior work linking p53 reactivation to enforced senescence and growth suppression. For example, in HPV-positive cancers, disruption of the HPV E6–p53 interaction restored p53, induced senescence, and suppressed tumor growth^60^. Similar oncogene-addiction mechanisms have been described in c-MYC-dependent lymphomas, where senescence escape arises through mutations or rearrangements restoring c-MYC activity^38^, while non-degradable c-MYC mutants sustain cell cycle re-entry^24^. In our model, Mdm2 upregulation likely substitutes for TagLuc by suppressing p53 and enabling escape. Interestingly, in other models it could be shown that Mdm2 inhibitors attenuate SASP factors^61^. This dual action, oncogenic suppression and SASP modulation, supports Mdm2 inhibition as a potential therapeutic approach. Nevertheless, our findings are most directly relevant to tumors that retain a functional p53 pathway but acquire Mdm2 overexpression or amplification, a genetic configuration present in several human malignancies. By contrast, in the many human cancers with *TP53*-mutations, OIIS and its escape are likely governed by p53-independent senescence programs and alternative forms of oncogene addiction, therefore Mdm2 inhibition would not be expected to provide the same therapeutic benefit. Whether analogous oncogene-addiction–like pathways are restored in relapses of *TP53*-mutant tumors, and whether such alternative senescence-escape circuits can likewise be leveraged therapeutically, will need to be addressed by future studies.

Persistent SASP activation fosters a pro-inflammatory, pro-angiogenic niche that supports regrowth^11,17^. Indeed, analysis of the tumor microenvironment across progression, remission, and relapse revealed marked remodeling of stromal and immune compartments. Relapsed tumors showed increased endothelial cells, suggesting neovascularization, and a shift toward an immunosuppressive milieu characterized by reduced dendritic cells and enriched regulatory macrophages. Comparable remodeling has been reported in TIS models, where SASP factors recruit Gr-1⁺ myeloid cells or polarize macrophages toward M2-like states^51,62,63^. These cells could either blunt immune surveillance or support neoangiogenesis. Our findings show that OIIS similarly reprograms the microenvironment, highlighting that relapse is not solely cell-intrinsic but emerges from co-evolution with the niche. In support of this concept, a recent study combining a next-generation pan-RAS(ON) inhibitor with CDK4/6 inhibitors in experimental PDAC models suggested that therapy-induced senescence can lead to durable tumor control via a senescence-associated immune equilibrium, even though the question, whether tumor-reactive T cells were involved, remained open^26^.

In conclusion, our study reveals that OIIS primes tumors for relapse through polyploidy, chromosomal instability, the acquisition of alternative oncogenic pathways, and SASP-driven microenvironmental remodeling. By characterizing the genetic, metabolic, cytogenetic, and immunological hallmarks of OIIS, we establish it as a distinct form of cellular state that initially constrains tumor growth but ultimately promotes progression, thereby providing a framework for identifying vulnerabilities and risks relevant to preventing cancer relapse.

## Methods

All animal experiments were performed according to national guidelines and approved by the Landesamt für Gesundheit und Soziales (Berlin, Germany). In vitro experiments were performed using established cell lines and did not require separate ethical approval.

### Cell culture and oncogene inactivation

Clone 4 cells and TTC #3055 cells were cultured in Dulbecco’s modified Eagle medium (DMEM, Gibco), supplemented with 10% heat inactivated fetal calf serum (PAN, Biotech) and 50 μg/ml gentamicin (Gibco). Clone 4 and TTC #3055 cells were cultured with 1 μg/ml doxycycline (Sigma) and medium was changed every 3 days. For oncogene inactivation, clone 4 and TTC #3055 cells were cultured, if not otherwise specified, in dox-free medium for 14 days. A375 melanoma cells were cultured in Dulbecco’s modified Eagle medium (DMEM, Gibco), supplemented with 10% heat inactivated fetal calf serum (PAN, Biotech) and 1× penicillin-streptomycin (Thermo Fisher Scientific). Cells were treated with 1 or 10 μM vemurafenib (TEBU-Bio, in DMSO) for the timepoints indicated in each figure and depending on the experiment, as specified in the corresponding figure legend. Medium was changed every 2 days.

### Western blot

Cells were lysed using the mammalian cell lysis kit (Sigma-Aldrich) or the RIPA buffer (Cell Signaling Technology) supplemented with protease/phosphatase inhibitor cocktail (Cell Signaling Technology). Protein concentrations were determined using the BCA assay (Pierce). Proteins were separated by SDS-PAGE gels and transferred to nitrocellulose membranes (Amersham/Bio-Rad). Primary antibodies included anti-SV40 T antigen (clone PAb416, Calbiochem, dilution: 1:1000), p16 INK4A (clones D7C1M and E5F3Y, Cell Signaling Technology, dilution 1:1000), p21 Waf1/Cip1 (clone E2R7A, Cell Signaling Technology, dilution 1:1000) and pRb (clone D20B12, Cell Signaling Technology, dilution 1:1000). For detection, membranes were incubated with either HRP-conjugated secondary antibodies (Southern Biotech, USA, dilution 1:10.000) and visualized using the SuperSignal chemiluminescent substrate kit (Thermo Fisher) on a LUMI-F1 workstation (Roche), or with IRDye 800CW goat anti-mouse IgG and IRDye 680RD goat anti-rabbit IgG (LI-COR Biosciences, dilution 1:10.000) and imaged using the LI-COR Odyssey system. Loading controls were performed using anti-β-actin antibodies (polyclonal, Abcam; monoclonal clone AC-15, Sigma-Aldrich; dilution 1:5000).

### SA-β-gal staining

Clone 4 cells were cultured in 6-well plates with or without doxycycline until they reached ~50–80% confluency. A375 cells were seeded at 15,000 cells per well and treated with 1 μM vemurafenib or vehicle (DMSO). Because of their different growth rates, SA-β-gal staining was performed on day 5 for vemurafenib-treated cells and day 2 for DMSO controls. Senescence-associated β-galactosidase activity was detected using either the Senescence Detection Kit (BioVision) or the SA-β-gal Staining Kit (Cell Signaling Technology). In both cases, cells were fixed with the provided fixation solution, washed, and incubated with X-gal staining solution at 37 °C overnight in a dry incubator (no CO₂). After staining, cells were stored in 70% glycerol at 4 °C until imaging. Stained cells were visualized using either an Olympus FSX100 microscope or a DMi8 inverted microscope (Leica Microsystems, 10× objective, scale bar 100 µm) to evaluate SA-β-gal activity.

### Cell cycle analysis

Cells were incubated with 10 μM BrdU (Sigma) for 60 min. Next, cells were washed with PBS and harvested. For permeabilization, cells were resuspended in ice-cold 70% ethanol mixed with PBS and stored for 2 h at −20 °C. Cells were washed again with PBS, incubated with 2 ml 2 N HCl/0,5 % Triton X-100 for 30 min, centrifuged and incubated with 2 ml 0.1 M sodium tetraborate (Sigma) for 10 min. After 2 washing steps with PBS, cells were stained with anti-BrdU antibody (Biolegend, 5 µl per 1 × 10⁶ cells in 100 µl staining volume) supplemented with 0,5% Tween-20 and 1% BSA for 30 min at room temperature. Next, cells were incubated with 20 μg/ml propidium iodide (containing 0,5% Tween-20, 1% BSA and 10 μg/ml RNase A (Qiagen) for 30 min at room temperature. Probes were analyzed via flow cytometry (BD FACS Canto II) and data was analyzed using FlowJo v10.8 Software (BD Life Sciences).

### Proliferation assay

A375 melanoma cells were seeded at 2000 cells per well into 96-well plates, with separate plates prepared for each time point. On day 0, cells were treated with either vehicle (DMSO) or 1 µM vemurafenib. Proliferation was assessed daily from day 0 to day 4 using the CellTiter 96 AQueous Non-Radioactive Cell Proliferation Assay (MTS; Promega) according to the manufacturer’s instructions. Briefly, the MTS/PMS reagent was added to each well and incubated for 1 h at 37 °C, after which absorbance was measured at 490 nm using a microplate reader. Each condition was plated in triplicate wells, and background absorbance from blank wells was subtracted. For each experiment, the mean absorbance of triplicates per condition was calculated at each time point. The experiment was independently repeated three times on different days.

### Real-time quantitative PCR

Total mRNA was extracted from proliferating, TagLuc expressing and senescent, TagLuc non-expressing clone 4 cells using the RNeasy Mini Kit (Qiagen). 1 μg was reverse-transcribed using an oligo-dT primer and ProtoScript II Reverse Transcriptase (NEB) Kit according to the manufacturer’s instructions. The reaction products were diluted (10x) in nuclease free water. 2 μl of the sample was subjected to real-time PCR, which was performed in triplicates using TaqMan probes for the mouse genes *CDKN1A*, *CDKN2A* and *GAPDH* (Applied Biosystems). Expression levels of *CDKN1A* and *CDKN2A* relative to that of *GAPDH* were calculated using the 2^−ΔΔCT^ method. The difference of the cycle threshold (CT) of the target and the reference gene was calculated (ΔCT) and compared between the two samples (ΔΔCT). Finally, the expression ratio was calculated according to the formula x = 2^−ΔΔCT^.

### Cytokine antibody array

Proliferating, TagLuc expressing and senescent, TagLuc non-expressing clone 4 cells were cultured in DMEM for 48 h to generate conditioned medium (CM). Cells were counted and CM was filtered and analyzed using Mouse Cytokine Antibody Array C2000 (RayBiotech) according to the manufacturer’s protocol. Briefly, array membranes were preincubated with 2 ml of blocking solution. CM was diluted with DMEM to reach volume equivalents of 2 × 10^5^ cells/ml. Array membranes were incubated with 1 ml of CM (3 h, room temperature), washed, and incubated with biotin-conjugated antibody cocktail (overnight, 4 °C). The next day, HRP-Streptavidin was added (2 h, room temperature), followed by a washing step of the array membranes and addition of the detection solution. Chemiluminescence was analyzed using the Lumi Imager F1 (Roche) with an exposure time of 1 min.

### ELISA

For the mouse system, conditioned medium (CM) was generated as described above, diluted in DMEM to a volume equivalent of 2 × 10⁵ cells/ml, and analyzed using IL-6 and MMP-3 ELISA kits (Life Technologies). For the human system, supernatants were directly collected from A375 melanoma cells treated with vemurafenib (1 µM, 15 days) or left untreated, and analyzed using the corresponding human IL-6 and MMP-3 ELISA kits (Life Technologies). All assays were performed according to the manufacturer’s instructions, and absorbance was measured using a µQuant microplate reader (BioTek).

### Luciferase activity assay

Cells were cultured in a 6-well plate, washed with PBS once and incubated with 500 μl of reporter lysis buffer (Promega) for 2 min. A cell scraper was used to harvest the cells and the cell suspension was transferred into Eppendorf tubes. The probes were snap-frozen in liquid nitrogen for 2 min, thawed on ice, and centrifuged at 12.000 rpm for 10 min. Protein concentration of the supernatant was determined by NanoDrop (Thermo Scientific). 10 μl of supernatant was mixed with luciferase assay buffer (Promega) and luciferase substrate buffer (Promega), respectively. TagLuc activity was measured using a Mithras LB 940 luminometer (Berthold Technologies) and relative light unit (RLU) was calculated according to total μg protein.

### RNA sequencing and differential expression analysis

RNA from proliferating (TagLuc expressing, +dox) and senescent (TagLuc non-expressing, -dox) cancer cells and cancer cells from relapsed tumors was isolated using the RNeasy Mini Kit (Qiagen). Replicate samples were generated from independently thawed frozen aliquots, expanded in separate flasks and processed independently for RNA extraction. mRNA integrity was measured using the Agilent TapeStation 4200. Libraries were sequenced on an HiSeq4000 platform. Differential gene expression analysis was performed as described before (detailed methods can be found in references^64,65^). In short, sequencing reads were quality-checked and mapped to the mouse genome. Differential expression analysis was performed using the DESeq2 R statistical package. For data analysis, the adjusted *p* value was set to <0.001 and the minimum log fold change of upregulated genes was set to ≥1. All procedures were performed in triplicates.

### Biological term enrichment analysis

Biological term enrichment analysis was carried out using two different methods. gProfiler2 R package (version 0.2.2) was used to detect enriched pathways (annotated as in the KEGG and Reactome databases) or GO terms among significantly upregulated/downregulated genes^66^. As a complementary approach, gene-set enrichment analysis was carried out using the fgsea R package (version 1.24.0) with cancer state signature genes downloaded from the CancerSEA database^34,67^. For the GSEA analysis, the genes were ranked by decreasing order of the -log10(*p*-value) multiplied by the sign of the log2-fold-change of each gene. For the results depicted in Supplementary Fig. 4A-B, enrichment of curated gene sets among differentially expressed genes was assessed using a one-tailed hypergeometric test. Up- and down-regulated genes were analysed against the background of all genes in the collection. For each set, the overlap with differentially expressed genes was counted, hypergeometric *p*-values and odds ratios were computed, and multiple testing correction was applied (FDR, Benjamini–Hochberg). Redundant sets with >90% overlap were pruned, and results were visualized by odds ratio and adjusted *p*-value, with top sets labeled.

### Oxygen consumption rate and extra-cellular acidification rate measurements

The oxygen consumption rate (OCR) and extra-cellular acidification rate (ECAR) were measured using a XF96 extracellular flux analyzer (Seahorse Bioscience). Cells were seeded at a density of 8 × 10^4^ (on dox) and 4 × 10^4^ (off dox) cells per well on poly-D-lysine (PDL)-treated plates, each well containing 200 µl of Seahorse XF Base Medium supplemented with 1 mM pyruvate, 2 mM glutamine, and 10 mM glucose. The plates were then incubated in a non-CO_2_ incubator at 37 °C for 45 min prior to the assay. The optimal cell density to maintain OCR within an optimal range for both conditions was determined experimentally. Basal mitochondrial respiration was assessed by subtracting the non-mitochondrial OCR, measured with 0.5 μM antimycin A and 0.5 μM piericidin A, from baseline OCR. Coupled respiration was determined by subtracting the OCR in the presence of 1.5 μΜ oligomycin A (Sigma) from the basal mitochondrial respiration. Maximal respiration was determined by subtracting the OCR in the presence of 1 μΜ FCCP (Sigma), from baseline OCR. The XF Glycolytic Rate Assay Kit was employed to measure basal and compensatory glycolysis. Basal measurements of extracellular acidification rate (ECAR) were followed by the sequential injection of 0.5 μM rotenone/antimycin A and 50 mM 2-DG. This allowed for the calculation of the glycolytic proton efflux rate (glycoPER), representing both basal glycolysis (in the absence of mitochondrial CO_2_ contribution) and compensatory glycolysis (as a result of electron transport chain inhibition). OCR and glycoPER values were normalized to 2 × 10^4^ cells to facilitate comparison across conditions. Data analysis was performed using the Seahorse Wave desktop software.

### Animal experiments

Mouse strains were housed under specific pathogen-free conditions at the animal facility of the Max Delbrück Center for Molecular Medicine under a 12-h light/12-h dark cycle. For cancer cell transplantation, proliferating or senescent cancer cells were harvested using 0.05% trypsin (Gibco), washed, resuspended in PBS, and mixed with Matrigel (BD, final concentration 10 mg/ml). The suspension was kept on ice until injection. Cells (1 × 10⁵) were inoculated subcutaneously (s.c.) into the flanks of mice using a 27-gauge needle (Braun). For oncogene activation, doxycycline (200 μg/ml dox, Sigma) was administered in light-protected drinking water supplemented with 5% sucrose. To verify TagLuc expression and exclude leakiness in the absence of doxycycline, luminescence imaging was performed in vitro prior to transplantation, and in vivo bioluminescent imaging (BLI) was carried out as described previously^28^. For the human xenograft experiments, 1 × 10⁵ A375 melanoma cells that had been pretreated in vitro with vemurafenib (1 µM, 14 days) to induce senescence were injected s.c. into Rag2-KO mice. Vemurafenib (TEBU-Bio) was prepared in PBS/PEG300 (7:3), stored at 4 °C, and administered intraperitoneally (25 mg/kg body weight). For the experiments reported here, mice additionally received intraperitoneal injections of the PBS/DMSO (25:1) vehicle, as they have been part of a therapy control group. The maximal tumor burden permitted by the approved protocol was an average tumor size of 15 mm × 15 mm × 15 mm, and this limit was not exceeded. Mice were monitored regularly for tumor growth, body condition, and general health status throughout the experiments. Disease progression was assessed by caliper-based tumor measurements and, where applicable, by in vivo bioluminescence imaging. Experiments were terminated either at predefined experimental time points or when humane endpoint criteria were reached, in accordance with the approved animal protocol. Humane endpoint criteria included excessive tumor burden, ulceration, impaired mobility, marked deterioration of general condition, or other signs of distress requiring euthanasia. 8 to 42 weeks old mice of both sexes were used for experiments. Sex was not specifically considered in the study design or analysis, as no sex-specific or hormonal effects on tumor growth were expected in this particular experimental setting.

### Spectral flow cytometry

Subcutaneous tumor plugs were isolated and digested for 1 h at 37 °C with shaking in a solution of RPMI 1640 medium (Gibco) and collagenase A (1.25 mg/ml). The digested tumors were filtered through a 70 µm cell strainer using the plunger of a 5 ml syringe and resuspended in FACS buffer (2% FSC in PBS). After tumor digestion, cells were counted using Countess 3 (Invitrogen). One million cells were transferred into a 96 well U bottom plate, centrifuged 5 min at 350 g and stained with the surface marker panel master mix using FACS buffer and Brilliant Stain buffer (BD) according to manufacturer’s instructions. Cells were stained at 4 °C for 30 min, followed by washing with FACS buffer, centrifugation for 5 min at 350 g and resuspension in 100 µL 2% PFA PBS for 15 min at room temperature. Cells were washed with FACS buffer, centrifuged for 5 min at 350 g and resuspended in 200 µL FACS buffer for flow cytometric analysis with the Cytek Aurora (Cytek Biosciences). One mouse sample had to be excluded from the analysis due to clogging.

Flow cytometry data were processed as described previously^68^. In brief, raw flow cytometry data were spectrally unmixed using the inbuilt unmixing function of the SpectroFlo (Cytek Biosciences) software. FCS files were imported into FlowJo (BD), parameters for generalized bi-exponential transformation of data were defined for every surface marker individually, and PeacoQC was used as an automatic quality control tool^69^. Live cells were exported using channel values defined by the inbuilt export function of FlowJo, imported into R, and processed with PICtR^68^. In short, data was subsampled per experimental group using the atomic sketching approach implemented in Seurat^70^, clustered using Louvain clustering^71^, and cells were annotated based on their feature expression. Cell type labels were predicted for all cells in the dataset using Linear Discriminant Analysis. Doublets were excluded from the analysis.

A summary of all antibodies used and the corresponding software for spectral flow cytometry is provided in Supplementary Data 5. Gating strategy is provided in the Source Data file.

### Detection of antigen-specific T cells in the blood

Detection of Tag-specific TCR expression on peripheral blood T cells was achieved by incubation with MHC/peptide tetramers and specific monoclonal antibodies in 50 μl PBS for 15 to 30 min at 4 °C. The following antibodies and tetramers were used: monoclonal anti-mouse CD3ε (1:100, clone 145-2C11, Biolegend); monoclonal anti-mouse CD8a (1:100, clone 53 − 6.7, Biolegend); H-2Kb/ VVYDFLKL (Tag peptide IV) tetramer (1:1000, Biozol/MBL). Cells were washed once with PBS and resolved in 200 μl PBS before analysis using a FACS Canto II or Symphony A1 devices (BD). Erythrocyte lysis was performed with BD lysing solution prior to FACS measurement. BD lysing solution was added for 8 to 10 min followed by one wash with PBS. Data was analyzed with FlowJo software (Treestar, Ashland, Oregon, USA).

### Spectral Karyotyping (SKY)

Spectral Karyotyping was performed as previously described^72^. In brief, metaphase preparation was performed by treating the cells with colcemid for 60 min at a concentration of 0.035 μg/ml. Next, cells were incubated in 75 mM KCl for 20 min at 37 °C and fixed in a freshly prepared mixture of methanol/acetic acid (3:1) at room temperature. Cell suspension was dropped onto glass slides and SKY images of 8-10 randomly selected metaphase chromosomes per cell line were acquired by staining with a mixture of 5 fluorochromes. Images were captured using an DMRXA epifluorescence microscope (Leica GmbH, Wetzlar, Germany), HCX PL SAPO 63×/1.30 oil objective (Leica), SpectraCube system (Applied Spectral Imaging, Migdal HaEmek, Israel) and analyzed using SKYView imaging software (Applied Spectral Imaging).

### Statistics and reproducibility

All data were analyzed using Prism v10 (GraphPad software) or R v4.4.2. No statistical method was used to predetermine sample size and sample sizes were estimated based on prior experience and on effect sizes observed in previous comparable experiments. One mouse sample was excluded from spectral flow cytometry analysis due to clogging. Mice in which no tumor growth occurred after subcutaneous injection were excluded from further tumor-based analyses. Mice that died from causes unrelated to tumor disease were excluded from the respective analyses. In addition, individual tumor measurements were excluded in cases where they represented clear outliers within an otherwise consistent series of longitudinal measurements. Data are presented as mean values with error bars as indicated in the respective figure legends, including standard deviation (SD), standard error of the mean (s.e.m.), or 95% confidence intervals (CI). The experiments were not randomized. The investigators were not blinded to allocation during experiments and outcome assessment. The number of independent experiments, biological replicates, technical replicates, and animals is indicated in the corresponding figure legends. Statistical analysis of the data was conducted using t-test or analysis of variance (ANOVA). Data comparison with *p* values of ≤ 0.05 was considered statistically significant. (ns indicates not significant, *p* > 0.05; * *p* ≤ 0.05; ** *p* ≤ 0.01; *** *p* ≤ 0.001; **** *p* ≤ 0.0001)

### Reporting summary

Further information on research design is available in the Nature Portfolio Reporting Summary linked to this article.

## Supplementary information

Supplementary Information

Description of Additional Supplementary Files

Supplementary Data 1

Supplementary Data 2

Supplementary Data 3

Supplementary Data 4

Supplementary Data 5

Reporting Summary

Transparent Peer Review file

## Source data

Source Data

## Data Availability

Source data are provided as a Source Data file. RNA sequencing data are provided in the Supplementary Data. The raw sequence data reported in the manuscript have been deposited in the Genome Sequence Archive in National Genomics Data Center, China National Center for Bioinformation / Beijing Institute of Genomics, Chinese Academy of Sciences (GSA: CRA018187) that are publicly accessible at (https://ngdc.cncb.ac.cn/gsa/browse/CRA018187^73,74^). The CancerSEA dataset is openly accessible and was used for comparative analyses^34^. Source data are provided with this paper.

## Code availability

Analysis Code for spectral flow cytometry data can be found at (https://github.com/ViktoriaFl/tumor_reject_immune_infiltration/. 10.5281/zenodo.19331353^75^).
