# Supplementary material for: Oncogene inactivation-induced senescence facilitates tumor relapse
Source: Nat Commun. 2026 Jul 15;17:6244. doi: 10.1038/s41467-026-75021-9 (PMC13373164; doi:10.1038/s41467-026-75021-9)
Supplement: Supplementary file 1 — Supplementary Information [file 41467_2026_75021_MOESM1_ESM.pdf]

Supplementary Information

Title:

Oncogene inactivation-induced senescence facilitates tumor relapse

The submission includes the following supporting information files:

Supplementary Figure

- Supplementary Figure 1. Characterization of senescence and SASP profiles following oncogene inactivation
- Supplementary Figure 2. Transcriptional and metabolic changes in senescent versus proliferating cancer cells
- Supplementary Figure 3. TagLuc-independent growth of relapsed tumors
- Supplementary Figure 4. Relapsed tumors are transcriptionally distinct from parental proliferating and senescent cells
- Supplementary Figure 5. Immune infiltration in progressive tumors, remission and relapse

Supplementary Data

- Supplementary Data 1: Differentially expressed genes between TagLuc-expressing and non-expressing clone 4 cells
- Supplementary Data 2: Differentially expressed genes between relapsed tumors and clone 4
- Supplementary Data 3: Annotation of the cytokine array
- Supplementary Data 4: Chromosomal aberrations identified in the clone 4 cell line
- Supplementary Data 5: Antibodies and software used for Spectral Flow Cytometry

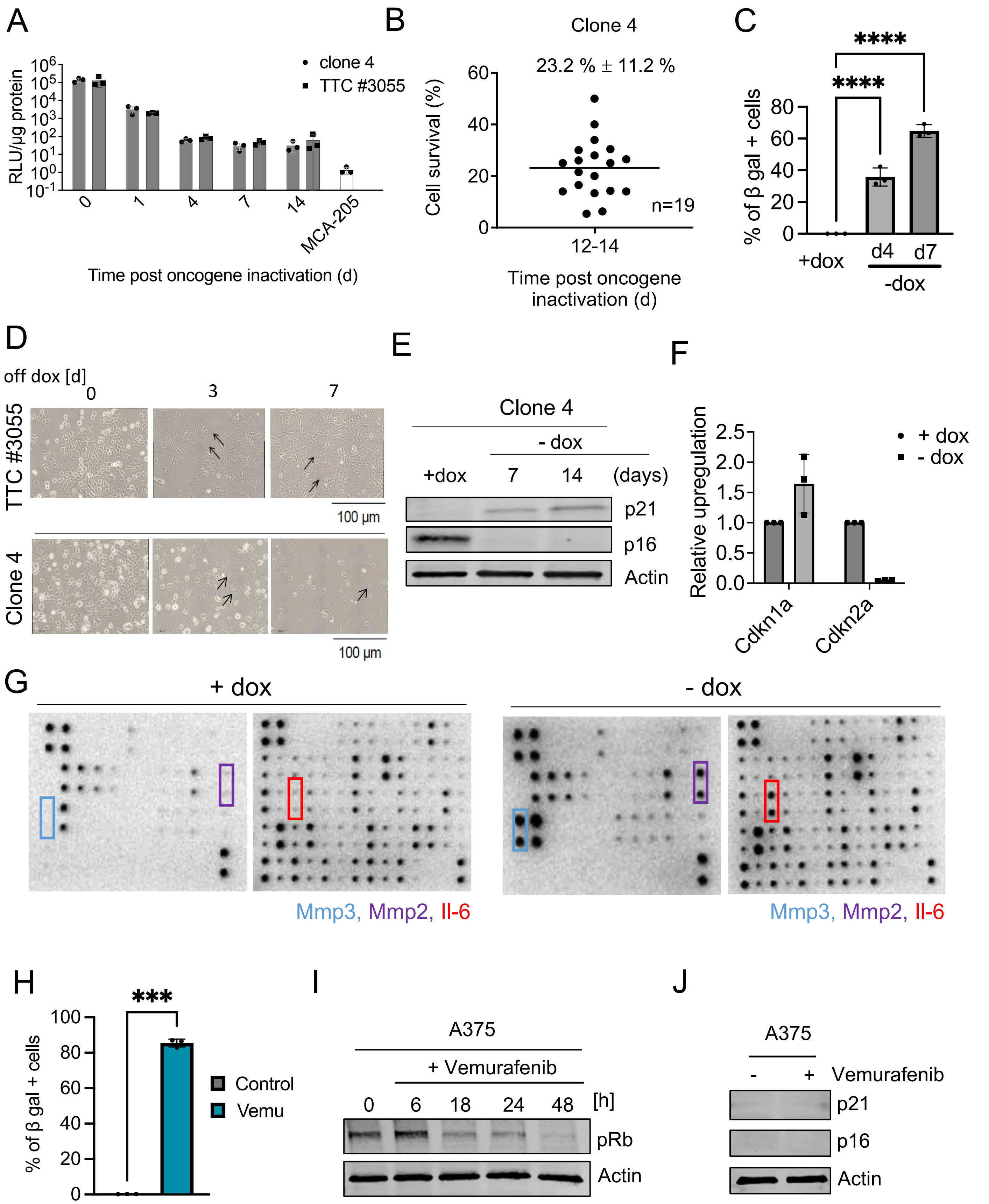
A
RLU/µg protein
clone 4
TTC #3055
0
1
4
7
14
MCA-205
Time post oncogene inactivation (d)
B
Clone 4
23.2 % ± 11.2 %
Cell survival (%)
n=19
12-14
Time post oncogene inactivation (d)
C
****
****
% of β gal + cells
+dox
d4
d7
-dox
D
off dox [d]
0
3
7
TTC #3055
Clone 4
100 µm
100 µm
E
Clone 4
- dox
+dox
7
14
(days)
p21
p16
Actin
F
Relative upregulation
+ dox
- dox
Cdkn1a
Cdkn2a
G
+ dox
- dox
Mmp3, Mmp2, Il-6
Mmp3, Mmp2, Il-6
H
***
% of β gal + cells
Control
Vemu
I
A375
+ Vemurafenib
0
6
18
24
48
[h]
pRb
Actin
J
A375
-
+
Vemurafenib
p21
p16
Actin

Supplementary Figure 1. Characterization of senescence and SASP profiles following oncogene inactivation.

A, Luciferase reporter activity in the presence of dox or at different time points following dox deprivation in clone 4 and TTC #3055 cells. Exposure time: 1s. Error bars indicate standard deviation; n=3 independent experiments. MCA-205 cells serve as negative control. **B,** The viability of clone 4 cancer cells was quantified 12 to 14 days after oncogene inactivation. The percentage of surviving clone 4 cells was calculated relative to the initial cell number before oncogene inactivation; n=19 independent experiments. **C**, Quantification of SA-β-galactosidase-positive cells after four or seven days post-TagLuc inactivation in clone 4 cells. Data acquired from three independent experiments. Error bars indicate standard deviation. ****p < 0.0001. **D,** Morphological changes of TTC#3055 and clone 4 cells were observed under phase-contrast light microscopy after growth on dox or at three- or seven-days post TagLuc inactivation. Enlarged and flattened cells with irregular shapes are indicated by black arrows. Data from one experiment are shown, although the observed phenotype was consistently observed. **E,** Western blot analysis of p21 and p16 protein levels in clone 4 cells grown on dox or after 7 or 14 days post-TagLuc inactivation. Actin was used as a loading control. Shown is one representative blot out of three independent experiments. The samples derive from the same experiment but different gels for p16 and p21 were processed in parallel. **F,** Levels of *CDKN1A* (p21) and *CDKN2A* (p16) mRNA relative to *GAPDH* mRNA in proliferating (TagLuc expressing, +dox) and senescent (TagLuc non-expressing, -dox) clone 4 cells measured by real-time PCR. Error bars indicate standard deviation; n=3 technical replicates from one experiment. **G,** Secretion of cytokines in proliferating (TagLuc expressing, +dox) and senescent (TagLuc non-expressing, -dox) cells. The experiment was conducted once. A comprehensive annotation of the array can be found in Supplementary Data 3. **H**, β-galactosidase staining of A375 cells after Vemu treatment. Percentage of positive cells is shown (mean ± s.d.; n = 3 independent experiments). *** p < 0,001. **I**, Western blot of phosphorylated Rb (pRb) in A375 cells treated with Vemu for the indicated times. Actin served as a loading control. Shown is one representative blot out of three independent experiments. **J,** Western blot showing the expression of the senescence

markers p21 and p16 after Vemu treatment. Shown is one representative blot out of three independent experiments. Actin served as a loading control. The samples derive from the same experiment but different gels for p16 and p21 were processed in parallel. Source data of this figure are provided as a Source Data file.

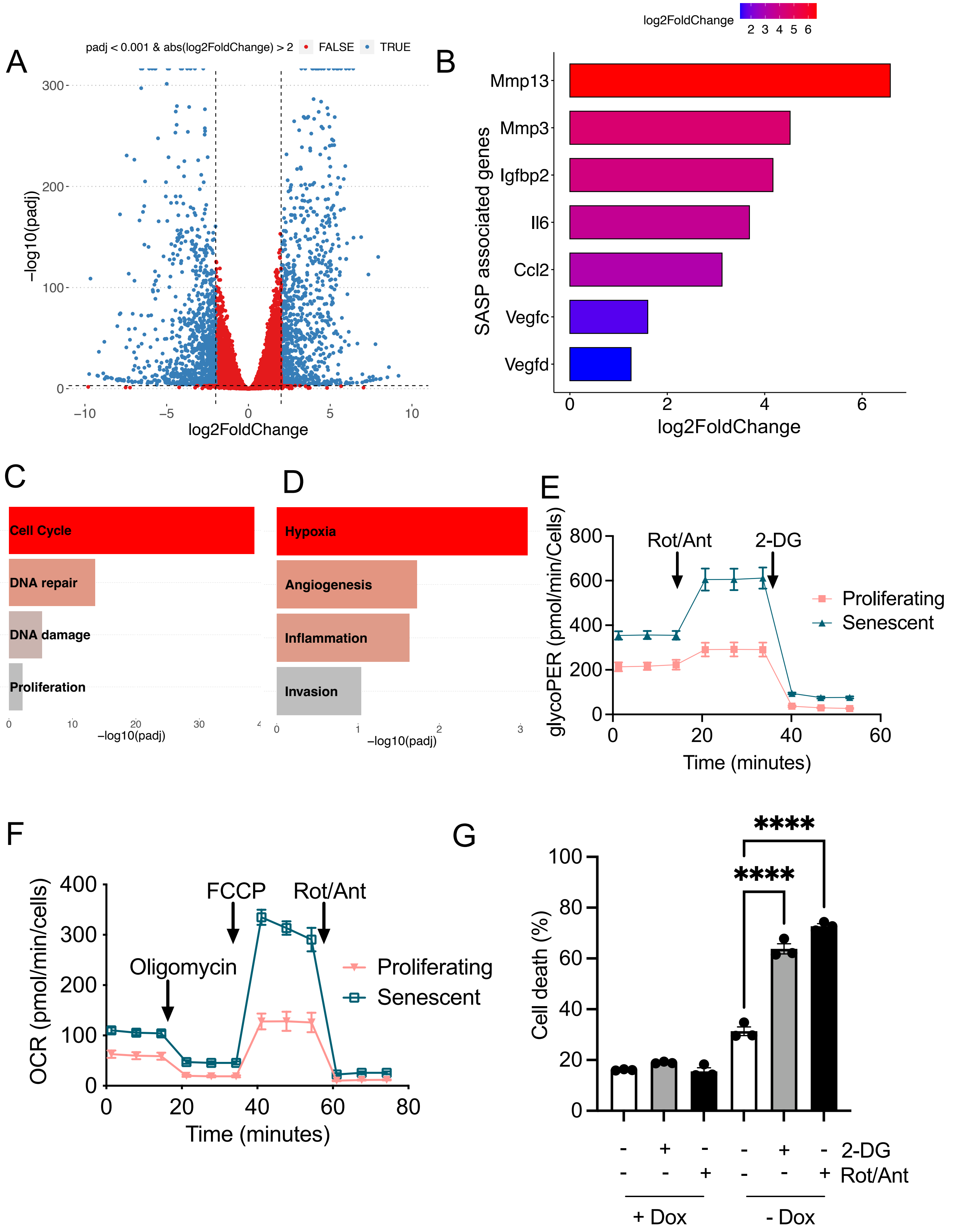
A
padj < 0.001 & abs(log2FoldChange) > 2 FALSE TRUE
−log10(padj)
log2FoldChange
B
log2FoldChange
Mmp13
Mmp3
Igfbp2
Il6
Ccl2
Vegfc
Vegfd
SASP associated genes
log2FoldChange
C
Cell Cycle
DNA repair
DNA damage
Proliferation
−log10(padj)
D
Hypoxia
Angiogenesis
Inflammation
Invasion
−log10(padj)
E
glycoPER (pmol/min/Cells)
Rot/Ant
2-DG
Proliferating
Senescent
Time (minutes)
F
OCR (pmol/min/cells)
Oligomycin
FCCP
Rot/Ant
Proliferating
Senescent
Time (minutes)
G
Cell death (%)
****
****
2-DG
Rot/Ant
+ Dox
- Dox

Supplementary Figure 2: Transcriptional and metabolic changes in senescent *versus* proliferating cancer cells.

A, Volcano plot showing the differentially upregulated and downregulated genes in senescent (TagLuc-negative, -dox) versus proliferating (TagLuc-expressing, +dox) cells. RNA-seq was performed on three independently processed replicate cultures. **B,** Differential expression of SASP-associated genes in senescent (TagLuc-negative, –dox) versus proliferating (TagLuc-expressing, +dox) cells. RNA-seq was performed on three independently processed replicate cultures. **C, D,** Gene-set enrichment analysis (GSEA) for CancerSEA cancer-state signatures downregulated (C) and upregulated (D) in senescent (TagLuc-negative, –dox) versus proliferating (TagLuc-expressing, +dox) cells. RNA-seq was performed on three independently processed replicate cultures. **E,** Representative profile of the extra-cellular acidification rate after addition of the complex I (rotenone) and complex III (antimycin) inhibitors and the glycolytic inhibitor (2-DG). Shown is mean ± SD, n = 8 technical replicates. **F,** Representative profile of the oxygen consumption rate after addition of the complex V inhibitor (oligomycin), the uncoupler (FCCP), and the complex I inhibitor (rotenone) and complex III inhibitor (antimycin) in proliferating (TagLuc-expressing, +dox) and senescent (TagLuc-negative, -dox) cells. Shown is mean ± SD, n = 5 technical replicates. **G,** Quantification of cell death in proliferating (+dox) and senescent (–dox, 7 days) cells after treatment with 2-DG (2 mM) or rotenone/antimycin A (R/A, 0.5 µM) for 48 h. Data are shown as mean ± s.e.m, n = 3 independent experiments. ****$p < 0.0001$. Source data of this figure are provided as a Source Data file.

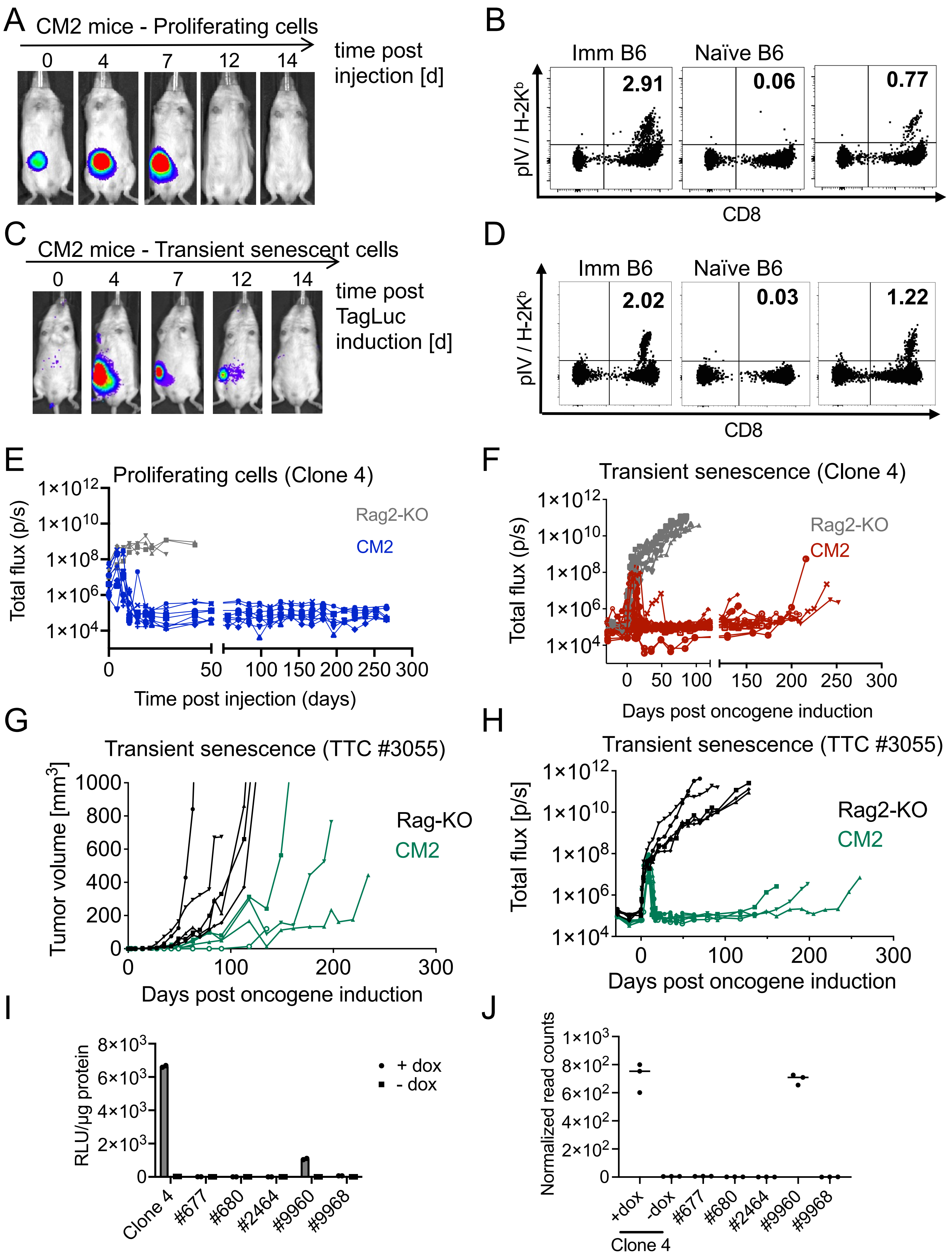

A
CM2 mice - Proliferating cells
0
4
7
12
14
time post injection [d]
B
Imm B6
Naïve B6
pIV / H-2Kb
2.91
0.06
0.77
CD8
C
CM2 mice - Transient senescent cells
0
4
7
12
14
time post TagLuc induction [d]
D
Imm B6
Naïve B6
pIV / H-2Kb
2.02
0.03
1.22
CD8
E
Proliferating cells (Clone 4)
Total flux (p/s)
Rag2-KO
CM2
Time post injection (days)
F
Transient senescence (Clone 4)
Total flux (p/s)
Rag2-KO
CM2
Days post oncogene induction
G
Transient senescence (TTC #3055)
Tumor volume [mm3]
Rag-KO
CM2
Days post oncogene induction
H
Transient senescence (TTC #3055)
Total flux [p/s]
Rag2-KO
CM2
Days post oncogene induction
I
RLU/µg protein
+ dox
- dox
Clone 4
#677
#680
#2464
#9960
#9968
J
Normalized read counts
+dox
-dox
Clone 4
#677
#680
#2464
#9960
#9968

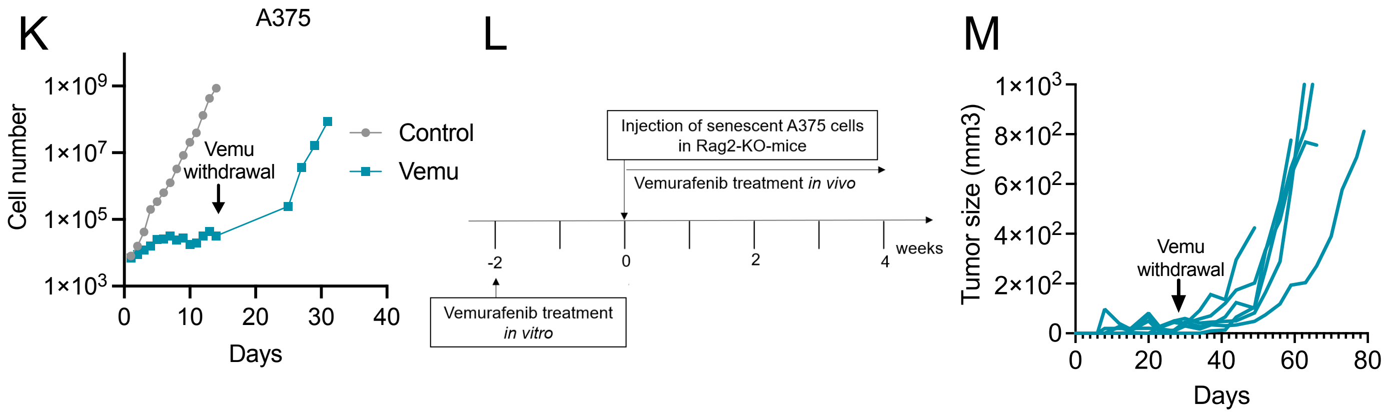

Supplementary Figure 3. TagLuc-independent growth of relapsed tumors.

A, Representative CM2 mouse inoculated with proliferating (TagLuc-expressing, +dox) clone 4 cells. TagLuc signal was monitored over time by BL imaging (exposure time: 60 s). **B,** Frequency of Tag-specific CD8$^+$ T cells in peripheral blood from immunized (Imm) B6, naïve B6, or tumor-bearing CM2 mice, measured 10 days after tumor injection. Representative dot plots are shown. Gating strategy is provided in the Source Data file. **C,** Representative CM2 mouse inoculated with senescent (TagLuc-negative, −dox) clone 4 cells. TagLuc signal was monitored over time by bioluminescence (BL) imaging (exposure time: 60 s) following oncogene reactivation. **D,** Frequency of Tag-specific CD8$^+$ T cells in peripheral blood from immunized C57BL/6 (Imm B6), naïve C57BL/6 (Naïve B6), or tumor-bearing CM2 mice, measured 12 days after oncogene re-activation. Representative dot plots are shown. Gating strategy is provided in the Source Data file. **E,** BL signal kinetics for Rag2-KO (n = 4) and CM2 (n = 8) mice inoculated with proliferating clone 4 cells (TagLuc-expressing, +dox). **F,** BL signal kinetics for immunodeficient Rag2-KO (n = 6) and immunocompetent CM2 (n = 13) mice inoculated with transient senescent (TagLuc-negative, −dox) clone 4 cells. The BL signal kinetics corresponds to the mice shown in Figure 3B. **G,** Tumor volume in Rag2-KO (n = 5) mice and relapse growth in CM2 (n = 6) mice following initial rejection of TTC #3055 cells. Two CM2 mice died before relapse assessment. **H,** BL signal kinetics for Rag2-KO (n = 5) and CM2 (n = 6) mice inoculated with transient senescent TTC #3055 cells (TagLuc-negative, −dox). **I,** Relative light units (RLU) per µg protein for parental clone 4 cells and tumor-derived cell lines from relapsed tumors, cultured ±dox. Four of five relapse-derived cell lines showed no detectable TagLuc activity, even in the presence of

dox. n = 2 technical replicates from one experiment. **J,** RNA sequencing of parental clone 4 and tumor cell lines from relapsed tumors. Normalized Tag read counts are shown for each sample. RNA-seq was performed on three independently processed replicate cultures. **K**, A375 cells were treated with 10 µM vemurafenib or left untreated, and cell numbers were monitored over time. Upon drug withdrawal (arrow), cells resumed proliferation. Viable cells were counted by trypan blue exclusion. Data from one experiment. **L**, Schematic of the *in vivo* experimental setup. Senescent A375 cells were injected into Rag2-KO mice and treated with Vemurafenib (Vemu). **M**, Tumor growth curves of Rag2-KO mice (n = 6) injected with senescent A375 cells and treated with Vemu according to the shown setup. Source data of this figure are provided as a Source Data file.

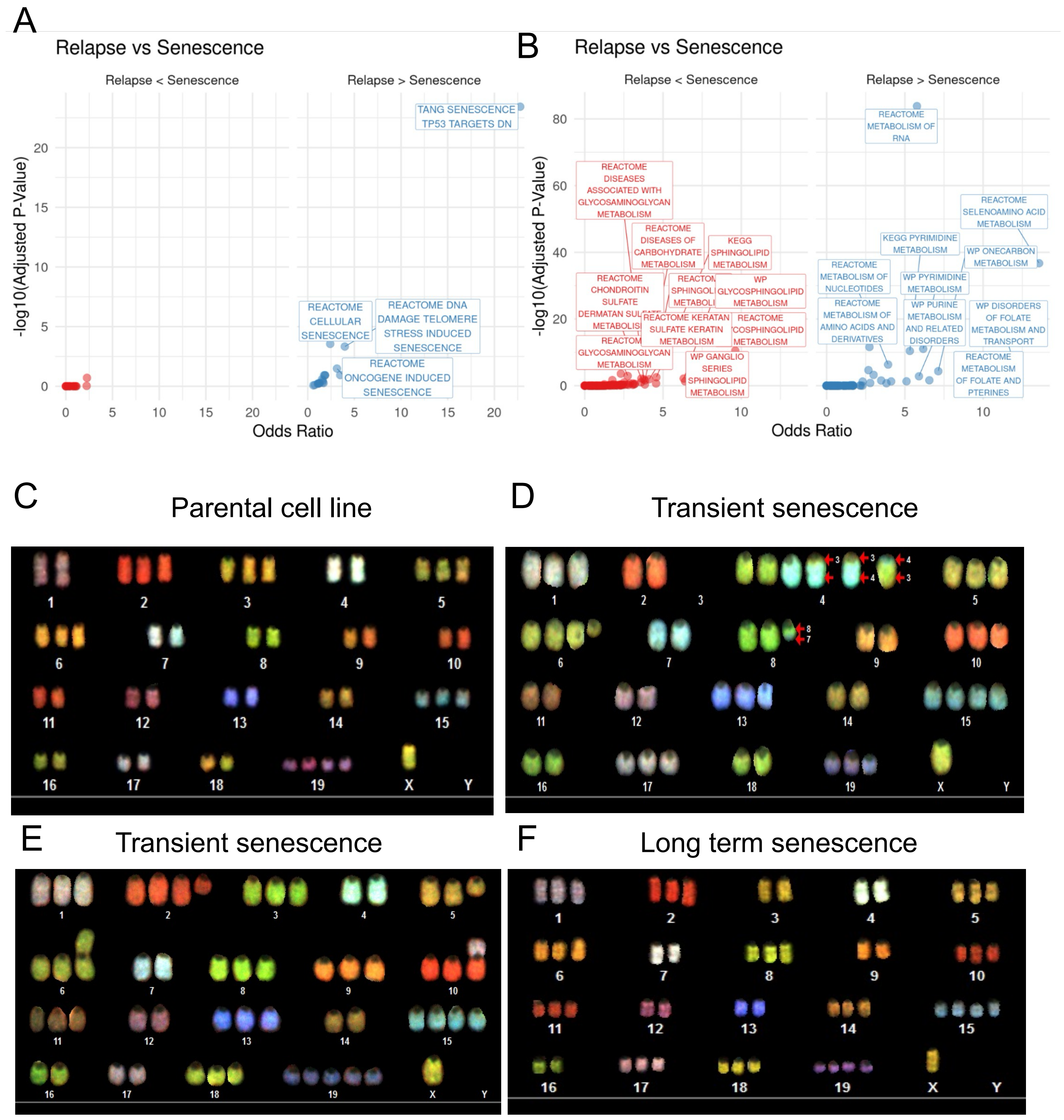

Supplementary Figure 4: Relapsed tumors are transcriptionally distinct from parental proliferating and senescent cells.

A, B, Top enriched senescence-related terms (A) and metabolism-related terms (B) detected among genes upregulated (“Relapse > Senescence”) or downregulated (“Relapse < Senescence”) in relapse-derived cancer cell lines compared to both parental proliferating (TagLuc-expressing, +dox) and senescent (TagLuc-negative, –dox) clone 4 cells. Pathway enrichment was based on

significantly differentially expressed genes (adjusted $p < 0.05$, $|\log_2 FC| > 1$). RNA-seq was performed on three independently processed replicate cultures. **C,** Representative spectral karyotyping (SKY) image of the parental clone 4 cell line maintained under continuous oncogene expression (+dox). **D-F,** Representative SKY images of relapse-derived cell lines from tumors that emerged after transient TagLuc inactivation (D, E) or long-term TagLuc inactivation (F). Images were obtained from 8–10 randomly selected metaphase spreads per cell line, with one representative example shown in each panel. Source data of this figure are provided as a Source Data file.

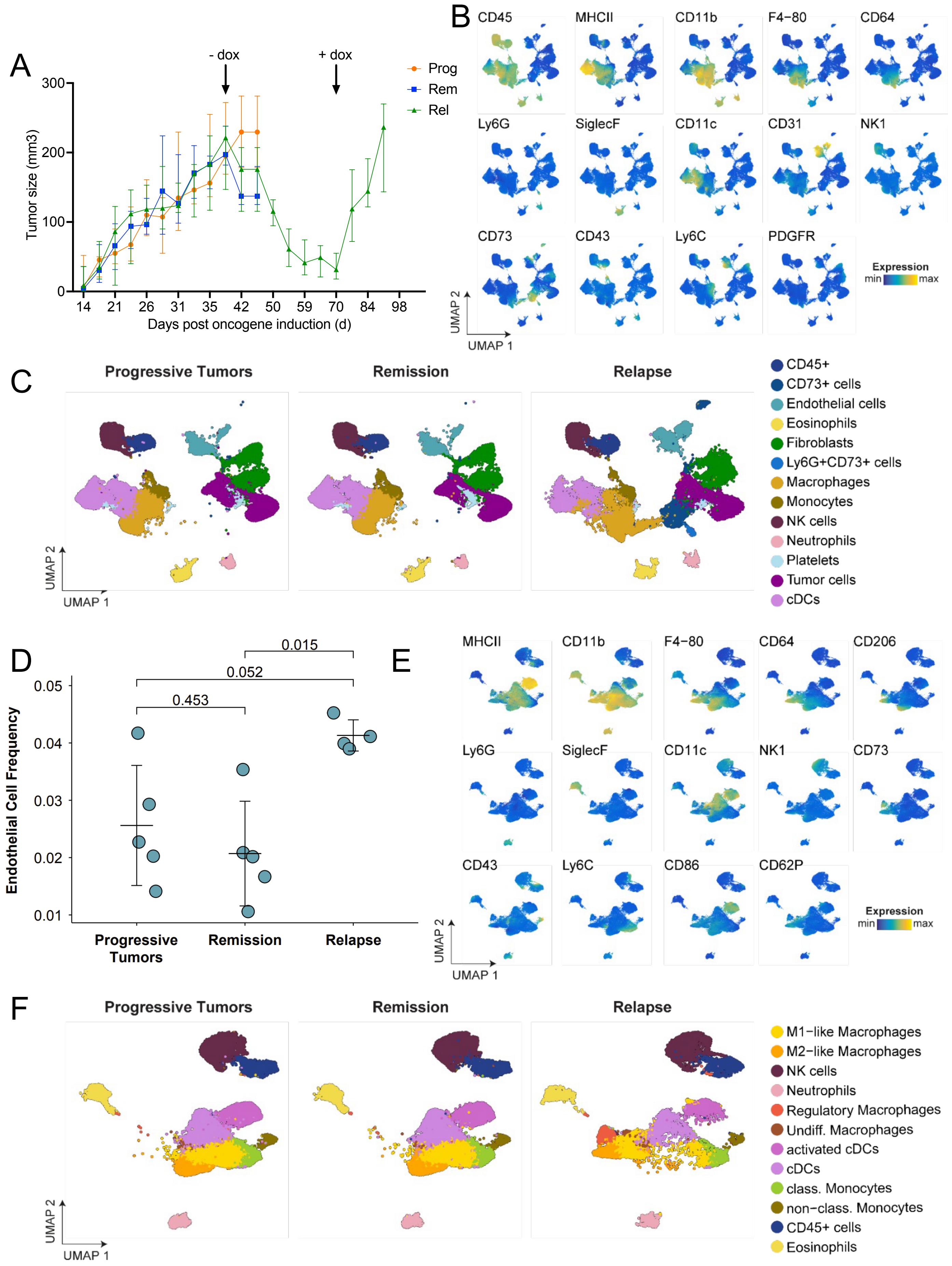

Supplementary Figure 5: Immune infiltration in progressive tumors, remission and relapse.

A, Tumor volume in progressive (Prog; n = 5 mice), remission (Rem; n =5 mice) and relapse (Rel;

n = 6 mice) groups depicted in Figure 5B. **B**, UMAP representations from Figure 5D, showing selected feature expression patterns for the overall cellular landscape. **C,** UMAP representation from Figure 5D, split by experimental group. **D,** Quantification of the endothelial cell frequency within all analysed cells across the experimental groups. *P* values were determined with a two-sided Welch's *t*-test and corrected according to Holm. Error bars indicate the mean and standard deviation. n = 5 biological replicates per group, one mouse sample from the relapse group was excluded from spectral flow cytometry analysis due to clogging. **E,** UMAP representations from Figure 5E, showcasing selected feature expression patterns for the immune cell annotation. **F,** UMAP representation of immune cells (see Figure 5E) split by experimental group. cDCs: conventional dendritic cells, NK cells: natural killer cells, non-class./class. Monocytes: non-classical/classical monocytes, UMAP: Uniform Manifold Approximation and Projection. Source data of this figure are provided as a Source Data file.
