# Supplementary material for: Oncogene inactivation-induced senescence facilitates tumor relapse
Source: Nat Commun. 2026 Jul 15;17:6244. doi: 10.1038/s41467-026-75021-9 (PMC13373164; doi:10.1038/s41467-026-75021-9)
Supplement: Supplementary file 2 — Description of Additional Supplementary Files [file 41467_2026_75021_MOESM2_ESM.pdf]

Title: Supplementary Data 1

Description: Differentially expressed genes between TagLuc-expressing and non expressing clone 4 cells.

Title: Supplementary Data 2

Description: Differentially expressed genes between relapsed tumors and clone 4.

Title: Supplementary Data 3

Description: Annotation of the cytokine array.

Title: Supplementary Data 4

Description: Chromosomal aberrations identified in the clone 4 cell line.

Title: Supplementary Data 5

Description: Antibodies and software used for spectral flow cytometry.
