# Supplementary material for: Oncogene inactivation-induced senescence facilitates tumor relapse
Source: Nat Commun. 2026 Jul 15;17:6244. doi: 10.1038/s41467-026-75021-9 (PMC13373164; doi:10.1038/s41467-026-75021-9)
Supplement: Supplementary file 8 — Reporting Summary [file 41467_2026_75021_MOESM8_ESM.pdf]

nature portfolio

Corresponding author(s): Philipp Schmitt, Inmaculada Martínez-Reyes, Thomas Blankenstein

Last updated by author(s): Mar 25, 2026

Reporting Summary

Nature Portfolio wishes to improve the reproducibility of the work that we publish. This form provides structure for consistency and transparency in reporting. For further information on Nature Portfolio policies, see our Editorial Policies and the Editorial Policy Checklist.

Statistics

For all statistical analyses, confirm that the following items are present in the figure legend, table legend, main text, or Methods section.

n/a	Confirmed	
☐	☒	The exact sample size (*n*) for each experimental group/condition, given as a discrete number and unit of measurement
☐	☒	A statement on whether measurements were taken from distinct samples or whether the same sample was measured repeatedly
☐	☒	The statistical test(s) used AND whether they are one- or two-sided<br>*Only common tests should be described solely by name; describe more complex techniques in the Methods section.*
☒	☐	A description of all covariates tested
☐	☒	A description of any assumptions or corrections, such as tests of normality and adjustment for multiple comparisons
☐	☒	A full description of the statistical parameters including central tendency (e.g. means) or other basic estimates (e.g. regression coefficient) AND variation (e.g. standard deviation) or associated estimates of uncertainty (e.g. confidence intervals)
☐	☒	For null hypothesis testing, the test statistic (e.g. *F*, *t*, *r*) with confidence intervals, effect sizes, degrees of freedom and *P* value noted<br>*Give P values as exact values whenever suitable.*
☒	☐	For Bayesian analysis, information on the choice of priors and Markov chain Monte Carlo settings
☒	☐	For hierarchical and complex designs, identification of the appropriate level for tests and full reporting of outcomes
☒	☐	Estimates of effect sizes (e.g. Cohen's *d*, Pearson's *r*), indicating how they were calculated

Our web collection on statistics for biologists contains articles on many of the points above.

Software and code

Policy information about availability of computer code

Data collection	For Flow cytometry, see below. Chemiluminescence was analyzed using the Lumi Imager F1 (Roche) and Mithras LB 940 luminometer (Berthold Technologies). For RNA-seq, mRNA integrity was measured using the Agilent Tape Station 4200 and probes were then analyzed via HiSeq4000. SKY Images were captured using an DMRXA epifluorescence microscope (Leica GmbH, Wetzlar, Germany), HCX PL SAPO 63×/1.30 oil objective (Leica), SpectraCube system (Applied Spectral Imaging, Migdal HaEmek, Israel). In vivo bioluminescent imaging (BLI) was performed using a Xenogen IVIS 200 device (Caliper Life Sciences, Waltham, Massachusetts, USA). The oxygen consumption rate (OCR) and extra-cellular acidification rate (ECAR) were measured using a XF96 extracellular flux analyzer (Seahorse Bioscience). For spectral flow cytometry, Cytek Aurora (Cytek Biosciences) was used. The western blot image adquisition was done using Odyssey CLx Imaging System (LICOR).
Data analysis	Prism v10 (GraphPad software) was used for statistical tests. Flow cytometry data was analyzed using FlowJo v10.8-10.10 Software (BD Life Sciences). For spectral flow cytometry, the inbuilt unmixing function of the SpectroFlo (Cytek Biosciences), FlowJo (BD, 10.10.0), PeacoQC (1.5.0) and R (R4.4.2) were used. SKY images were analyzed using SKYView imaging software (Applied Spectral Imaging). Living image software (version 2.6, Caliper Life Sciences) was used to analyze BL data. Differential expression analysis was performed using the DESeq2 R statistical package. Biological term enrichment analysis was carried out using gProfiler2 R package (version 0.2.2) and the fgsea R package (version 1.24.0) with cancer state signature genes downloaded from the CancerSEA database.

For manuscripts utilizing custom algorithms or software that are central to the research but not yet described in published literature, software must be made available to editors and reviewers. We strongly encourage code deposition in a community repository (e.g. GitHub). See the Nature Portfolio guidelines for submitting code & software for further information.

Data

Policy information about availability of data

All manuscripts must include a data availability statement. This statement should provide the following information, where applicable:

- Accession codes, unique identifiers, or web links for publicly available datasets
- A description of any restrictions on data availability
- For clinical datasets or third party data, please ensure that the statement adheres to our policy

Data availability:
Source data are provided as a Source Data file. RNA sequencing data are provided in the Supplementary Data The raw sequence data reported in the manuscript have been deposited in the Genome Sequence Archive in National Genomics Data Center, China National Center for Bioinformation / Beijing Institute of Genomics, Chinese Academy of Sciences (GSA: CRA018187) that are publicly accessible at https://ngdc.cncb.ac.cn/gsa. The CancerSEA dataset is openly accessible and was used for comparative analyses.

Code availability:
Analysis Code for spectral flow cytometry data can be found at https://github.com/ViktoriaFl/tumor_reject_immune_infiltration/. DOI: 10.5281/zenodo.19331353

Research involving human participants, their data, or biological material

Policy information about studies with human participants or human data. See also policy information about sex, gender (identity/presentation), and sexual orientation and race, ethnicity and racism.

Reporting on sex and gender	*Use the terms sex (biological attribute) and gender (shaped by social and cultural circumstances) carefully in order to avoid confusing both terms. Indicate if findings apply to only one sex or gender; describe whether sex and gender were considered in study design; whether sex and/or gender was determined based on self-reporting or assigned and methods used. Provide in the source data disaggregated sex and gender data, where this information has been collected, and if consent has been obtained for sharing of individual-level data; provide overall numbers in this Reporting Summary. Please state if this information has not been collected. Report sex- and gender-based analyses where performed, justify reasons for lack of sex- and gender-based analysis.*
Reporting on race, ethnicity, or other socially relevant groupings	*Please specify the socially constructed or socially relevant categorization variable(s) used in your manuscript and explain why they were used. Please note that such variables should not be used as proxies for other socially constructed/relevant variables (for example, race or ethnicity should not be used as a proxy for socioeconomic status). Provide clear definitions of the relevant terms used, how they were provided (by the participants/respondents, the researchers, or third parties), and the method(s) used to classify people into the different categories (e.g. self-report, census or administrative data, social media data, etc.) Please provide details about how you controlled for confounding variables in your analyses.*
Population characteristics	*Describe the covariate-relevant population characteristics of the human research participants (e.g. age, genotypic information, past and current diagnosis and treatment categories). If you filled out the behavioural & social sciences study design questions and have nothing to add here, write "See above."*
Recruitment	*Describe how participants were recruited. Outline any potential self-selection bias or other biases that may be present and how these are likely to impact results.*
Ethics oversight	*Identify the organization(s) that approved the study protocol.*

Note that full information on the approval of the study protocol must also be provided in the manuscript.

Field-specific reporting

Please select the one below that is the best fit for your research. If you are not sure, read the appropriate sections before making your selection.

☒ Life sciences ☐ Behavioural & social sciences ☐ Ecological, evolutionary & environmental sciences

For a reference copy of the document with all sections, see nature.com/documents/nr-reporting-summary-flat.pdf

Life sciences study design

All studies must disclose on these points even when the disclosure is negative.

Sample size	No statistical method was used to predetermine sample size and sample sizes were estimated based on prior experience and on effect sizes observed in previous comparable experiments.
Data exclusions	One mouse sample was excluded from spectral flow cytometry analysis due to clogging. Mice in which no tumour growth occurred after subcutaneous injection were excluded from further tumour-based analyses. Mice that died from causes unrelated to tumour disease were also excluded from the respective analyses. In addition, individual tumour measurements were excluded in cases where they represented clear outliers within an otherwise consistent series of longitudinal measurements.

Replication	All experimental data was reliably reproduced in multiple experiments as indicated in the figure legends. In vivo tumor experiments were performed one to three times. Number of independent experiments are specifised in the figure legend.
Randomization	Experimental animals were not randomized to experimental groups, but were age-matched, sex-matched, and littermates when possible.
Blinding	Investigators were not blinded.

Reporting for specific materials, systems and methods

We require information from authors about some types of materials, experimental systems and methods used in many studies. Here, indicate whether each material, system or method listed is relevant to your study. If you are not sure if a list item applies to your research, read the appropriate section before selecting a response.

Materials & experimental systems

n/a	Involved in the study	
☐	☒	Antibodies
☐	☒	Eukaryotic cell lines
☒	☐	Palaeontology and archaeology
☐	☒	Animals and other organisms
☒	☐	Clinical data
☒	☐	Dual use research of concern
☒	☐	Plants

Methods

n/a	Involved in the study	
☒	☐	ChIP-seq
☐	☒	Flow cytometry
☒	☐	MRI-based neuroimaging

Antibodies

Antibodies used

- anti-SV40 T antigen; clone PAb416; Calbiochem; application: Western blot
- anti-p16INK4A; clones D7C1M and E5F3Y; Cell Signaling Technology; application: Western blot
- anti-p21Waf1/Cip1; clone E2R7A; Cell Signaling Technology; application: Western blot
- anti-β-actin; clone AC-15; Sigma-Aldrich; application: Western blot
- HRP-conjugated secondary antibodies; Southern Biotech; application: Western blot
- IRDye 800CW goat anti-mouse IgG; LI-COR Biosciences; application: Western blot
- IRDye 680RD goat anti-rabbit IgG; LI-COR Biosciences; application: Western blot
- anti-pRb; clone D20B12; Cell Signaling Technology; application: Western blot
- anti-BrdU; BioLegend; application: Flow cytometry
- anti-mouse CD3ε; clone 145-2C11; BioLegend; application: Flow cytometry
- anti-mouse CD8a; clone 53–6.7; BioLegend; application: Flow cytometry
- anti- H-2Kb/VVYDFLKL (Tag peptide IV) tetramer; Biozol/MBL; application: Flow cytometry
- anti-CD48; clone HM48-1; BD; application: Spectral Flow Cytometry
- anti-CD48; clone HM48-1; BD; application: Spectral Flow Cytometry
- anti-CD41; clone MWReg30; BD; application: Spectral Flow Cytometry
- anti-CD43; clone S7; BD; application: Spectral Flow Cytometry
- anti-CD86; clone GL1; BD; application: Spectral Flow Cytometry
- anti-CD16/32; clone 2.4G2; BD; application: Spectral Flow Cytometry
- anti-MHCII; clone M5/114.15.2; BD; application: Spectral Flow Cytometry
- anti-CD127; clone SB/199; BD; application: Spectral Flow Cytometry
- anti-Siglec F; clone 1RNM44N; Thermo Fisher; application: Spectral Flow Cytometry
- anti-CD206; clone MR5D3; BioRad; application: Spectral Flow Cytometry
- anti-CD62P; clone RB40.34; BD; application: Spectral Flow Cytometry
- anti-CD71; clone RI7217; BioLegend; application: Spectral Flow Cytometry
- anti-NK1.1; clone PK136; BioLegend; application: Spectral Flow Cytometry
- anti-CD64; clone X54-5/7.1; BioLegend; application: Spectral Flow Cytometry
- anti-CD23; clone B3B4; BD; application: Spectral Flow Cytometry
- anti-CD117; clone 2B8; BioLegend; application: Spectral Flow Cytometry
- anti-CD80; clone 16-10A1; BD; application: Spectral Flow Cytometry
- anti-CD150; clone TC15-12F12.2; BioLegend; application: Spectral Flow Cytometry
- anti-CD105; clone MJ7/18; BD; application: Spectral Flow Cytometry
- anti-CD21/35; clone 7E9; BioLegend; application: Spectral Flow Cytometry
- anti-Ly6C; clone HK1.4; BioLegend; application: Spectral Flow Cytometry
- anti-CD11b; clone M1/70; BD; application: Spectral Flow Cytometry
- anti-CD31; clone 390; BD; application: Spectral Flow Cytometry
- anti-F4/80; clone W20065D; BioLegend; application: Spectral Flow Cytometry
- anti-CD135; clone A2F10.1; BD; application: Spectral Flow Cytometry
- anti-CD11c; clone N418; BioLegend; application: Spectral Flow Cytometry

- anti-CD73; clone TY/11.8; Thermo Fisher; application: Spectral Flow Cytometry
- anti-PDGFR-alpha; clone APA5; BioLegend; application: Spectral Flow Cytometry
- anti-B220; clone RA3-6B2; BioLegend; application: Spectral Flow Cytometry
- anti-Ly6G; clone 1A8; BD; application: Spectral Flow Cytometry
- anti-FAP; clone 983802; BioTechne; application: Spectral Flow Cytometry
- anti-CD34; clone RAM34; BD; application: Spectral Flow Cytometry
- anti-Sca1; clone D7; BD; application: Spectral Flow Cytometry
- anti-CD45; clone 30-F11; BioLegend; application: Spectral Flow Cytometry

Validation: The antibodies used in this study were tested by the manufacturer.

Eukaryotic cell lines

Policy information about cell lines and Sex and Gender in Research

Cell line source(s)	Clone 4 cells were generated from a gastric carcinoma,TTC#3055 from a spindle cell sarcoma isolated from the snout. A375 melanoma cells are commercially available.
Authentication	Neither of the cell lines used were authenticated.
Mycoplasma contamination	Cell lines were not tested for mycoplasma contamination.
Commonly misidentified lines (See ICLAC register)	These cell lines are not listed in the database of commonly misidentified cell lines maintained by ICLAC.

Animals and other research organisms

Policy information about studies involving animals; ARRIVE guidelines recommended for reporting animal research, and Sex and Gender in Research

Laboratory animals	All mouse strains were housed at the animal facility of the Max-Delbrück Center for Molecular Medicine under specific pathogen-free conditions. 8 to 42 weeks old mice of both sexes were used for experiments. Immunocompetent transgenic CM2 mice expressing the rtTA (Anders K. et al. Cancer Cell 2011), C57BL/6 or immunodeficient Rag2-KO mice were used.
Wild animals	This study did not involve wild animals.
Reporting on sex	Mice of both sexes were used but sex-based analysis was not performed as we did not expect an effect of sex on tumor growth.
Field-collected samples	This study did not involve samples collected from the field.
Ethics oversight	All animal experiments were performed according to national guidelines and were approved by the responsible national institute (Landesamt für Gesundheit und Soziales, Berlin, Germany).

Note that full information on the approval of the study protocol must also be provided in the manuscript.

Plants

Seed stocks	*Report on the source of all seed stocks or other plant material used. If applicable, state the seed stock centre and catalogue number. If plant specimens were collected from the field, describe the collection location, date and sampling procedures.*
Novel plant genotypes	*Describe the methods by which all novel plant genotypes were produced. This includes those generated by transgenic approaches, gene editing, chemical/radiation-based mutagenesis and hybridization. For transgenic lines, describe the transformation method, the number of independent lines analyzed and the generation upon which experiments were performed. For gene-edited lines, describe the editor used, the endogenous sequence targeted for editing, the targeting guide RNA sequence (if applicable) and how the editor was applied.*
Authentication	*Describe any authentication procedures for each seed stock used or novel genotype generated. Describe any experiments used to assess the effect of a mutation and, where applicable, how potential secondary effects (e.g. second site T-DNA insertions, mosiacism, off-target gene editing) were examined.*

Flow Cytometry

Plots

Confirm that:

- [ ] The axis labels state the marker and fluorochrome used (e.g. CD4-FITC).
- [x] The axis scales are clearly visible. Include numbers along axes only for bottom left plot of group (a 'group' is an analysis of identical markers).
- [ ] All plots are contour plots with outliers or pseudocolor plots.
- [x] A numerical value for number of cells or percentage (with statistics) is provided.

Methodology

Sample preparation	Sample preparation is described in detail in the methods section of the manuscript.
Instrument	- Cytek Aurora (Cytek Biosciences)<br>- BD FACSCanto II (BD)<br>- BD FACSymphony A1 (BD)<br>- FACScan flow cytometer (Becton Dickinson; Heidelberg, Germany)
Software	- SpectroFlo (Cytek Biosciences) – spectral unmixing<br>- FlowJo (BD, 10.10.0) – FCS import, transformation, gating/export<br>- PeacoQC (1.5.0) – automatic quality control<br>- R (R v4.4.2) – downstream processing<br>- PICtR (1.0.0)– downstream processing in R<br>- Seurat (5.3.0) – subsampling (atomic sketching) and downstream single-cell workflow<br>- CellQuest – acquisition/analysis software used with FACScan (FL-2 channel DNA content)<br><br>An overview of the corresponding software for spectral flow cytometry is also provided in the Supplementary Data.
Cell population abundance	Aliquoted tumor samples were regularly thawed to allow 100% purity of cancer cell analysis for flow cytometry. For tetramer staining, cells were seperated from cell debris and erythrocytes and other white blood cell populations.<br><br>Cell population abundance was reported as “frequency” of annotated cell types. Frequencies were derived after spectral flow cytometry processing and are specified in the method section. Total cell live count is additionally provided in the Source Data.
Gating strategy	Gating strategy for % of TET+ Cells:<br>SSC-A vs. FSC-A: Lymphocytes population<br>CD8 vs. CD3 (showing the Lymphocytes population): CD3+ T cell population<br>CD8 vs. TET-IV-PE (showing the T cell population): % of TET+ (antigen specific) population<br><br>For tetramer staining, immunized and naive C57BL/6 mice were used as positive/negative control to allow differentiation between positive/negative staining.<br><br>For spectral flow cytometry: data were spectrally unmixed using SpectroFlo (Cytek Biosciences), quality controlled with PeacoQC, and analyzed in FlowJo and R using PICtR. Cells were subsampled using atomic sketching (Seurat), clustered by Louvain clustering, and annotated based on marker expression. Cell type labels were assigned by Linear Discriminant Analysis after exclusion of doublets and dead cells.

- [x] Tick this box to confirm that a figure exemplifying the gating strategy is provided in the Supplementary Information.
