# Supplementary material for: Oncogene inactivation-induced senescence facilitates tumor relapse
Source: Nat Commun. 2026 Jul 15;17:6244. doi: 10.1038/s41467-026-75021-9 (PMC13373164; doi:10.1038/s41467-026-75021-9)
Supplement: Supplementary file 9 — Transparent Peer Review file [file 41467_2026_75021_MOESM9_ESM.pdf]

nature portfolio

Peer Review File

Oncogene inactivation-induced senescence facilitates tumor relapse

Corresponding Author: Mr Philipp Schmitt

This file contains all reviewer reports in order by version, followed by all author rebuttals in order by version.

Version 0:

Reviewer comments:

Reviewer #1

(Remarks to the Author)
This manuscript by Schmitt and colleagues has the potential to contribute to the literature relating to senescence and escape from senescence. The strengths of this work are the sophisticated experimental approaches utilized, the relative novelty of studies relating to senescence induced by oncogene inactivation rather than the more well-established Oncogene-induced Senescence (OIS), and the findings relating to the regulation of this form of senescence and escape from senescence. In addition, the findings that oncogene-inactivation is transient and can facilitate long-term relapse, and that oncogene-inactivated senescent cells are amenable to senolysis by autophagy inhibition in vitro (but not in vivo) may represent important contributions to the field. However, the work has a number of deficiencies. One is that oncogene-induced senescence and its relationship to the current work is entirely ignored. There is also insufficient discussion of other studies in the literature involving senescence and escape from senescence both in tumor bearing animals and disease recurrence in patients such as the seminal work of Duy et al. and Milanovic et al. There is also some concern that the findings are based on a single, specific, genetically-engineered model.

Overall Concerns
1. The authors use the dox-switchable on-off Tag oncogene tumor cell model in their analyses. Chronic dox treatment results in the activation of Tag, which then drives cellular hyperproliferation consistent with malignant transformation. This is often associated with the development of “oncogene addiction” which can lead to growth arrest once “inactivated”. This is similar to previously reported findings where the inactivation of an oncogene in oncogene- addicted cells can in fact lead to senescence: c-myc (PMID: 37656618; Afifi MM et al,. Irreversible cell cycle exit associated with senescence is mediated by constitutive MYC degradation) or HPV E7 (PMID: 31693922 Celegato M et al, A novel small-molecule inhibitor of the human papillomavirus E6-p53 interaction that reactivates p53 function and blocks cancer cells growth. Cancer Lett. 2020 Feb 1;470:115-125.) and PMID: 31603527). These previous findings must be considered in the formulation of a more in-depth interpretation of the data.

2. The dox-switchable on-off Tag oncogene tumor cell model allows for promotion or inhibition of the expression of Tag rather than activation/deactivation of the oncogene. The only way to confirm the primary proposal in this manuscript (i.e. that oncogene inactivation rather than activation is what drives senescence) is to directly (pharmacologically or genetically) inactivate oncogenes in various tumor cell models (such as RAS, B-Raf, and c-myc, among others). Inactivating one of these oncogenes in another cellular model would provide proof-of-concept and support the central proposition of the manuscript.

3. While the acute reduction in Tag expression clearly induces the expression of p21, this is also accompanied by p16 downregulation, which is unusual. The authors attempt to explain this observation by the assumption that Tag withdrawal-induced senescence is dependent on p21 and not p16; however, this hypothesis requires further experimental evaluation (e.g., knocking down either protein and observing the outcome on senescence induction in vitro).

There are also a number of assumptions and presumptions that are not sufficiently supported by experimental data, as delineated below:

Figure 1: This figure absolutely requires quantification of senescence, which is the fundamental observation underlying all subsequent studies. Images of beta galactosidase staining in the cells are insufficient. It is further insufficient to only measure expression (message levels) of p21 and p16; data must be provided relating to protein levels, as is consistent

throughout the literature. Finally, the authors refer to Rb as a critical factor relating to senescence induction, but neglect to assess levels of phosphorylated (inactive) and dephosphorylated Rb (the active form that is considered to be critical for promotion of senescence as well as levels of the Rb-E2F complex(es), where binding to the dephosphorylated form of Rb by E2F arrests the cell.

Figure 2. Were these data generated only from a single clone? This is insufficient to allow the authors or readers to draw conclusions relating to the broader relevance of these observations. The data must be confirmed by studies in another model of oncogene inactivation induced senescence. Moreover, transcriptomic analysis must consider the application of well-established senescence signatures such as Fridman's UP (which provides a comprehensive transcriptomic signature of gene expression and pathways indicative of senescence).

Figure 3. The conclusions relating to mdm2 and p53 are intriguing. Unfortunately, the authors have neglected to demonstrate that the pharmacological mdm2 inhibitor utilized in these studies has a direct impact on mdm2 and p53. Moreover, despite being state-of-the-art, studies in Figure 3 only provide proof that "transient" or "escapable" senescence can predispose to cancer relapse rather than providing a specific interpretation of this phenomenon with respect to oncogene-inactivation. These data are important and relevant to the proposal that tumor cell senescence can serve as a mechanism for cancer relapse, but only if more thoughtfully interpreted. Findings related to mdm2 dependence must be discussed in the light of PMID: 29402901 (Small molecule antagonists attenuate the senescence associated secretory phenotype).

Figure 5. The Y axis in Figure 5A is indicated to demonstrate "relative cell death". This is an inappropriate measurement and instead the authors must indicate the actual percentage of cells that die, as is the case for the apoptosis measurements. With respect to autophagy, which is a central component of the latter portion of the manuscript, the one assay that is utilized (LC3I and II levels) does not provide convincing data. Additional assays evaluating autophagy induction and suppression with both pharmacological and genetic autophagy inhibition must be provided to support any conclusions relating to the relationship between autophagy and senescence in these studies.

Figure 5. The results in the in vivo experiment presented in Figure 5E are striking in largely contradicting one of the primary concepts discussed in the latter section of manuscript, specifically that inhibition of autophagy would cull oncogene inactivation-induced senescence. An alternative explanation is that autophagy is likely to facilitate an immune response against senescent cells (or perhaps just tumor cells) which results in the observed contradictory effect in immunodeficient mice. In an effort to extend and confirm this observation, tumor-bearing animal studies relating to autophagy must also be provided utilizing cells where Beclin has been suppressed (as in the cell culture work).

Reviewer #2

(Remarks to the Author)
Manuscript Title: Oncogene inactivation-induced senescence facilitates tumor relapse
In the manuscript entitled "Oncogene inactivation-induced senescence facilitates tumor relapse", Schmitti et al. demonstrate that oncogene inactivation leads to the induction of cellular senescence and the establishment of a pro-inflammatory senescence-associated secretory phenotype (SASP) both in vitro and in vivo. Based on their findings, the senescent cells displayed an increased propensity for tumor relapse, since they acquired chromosomal abnormalities, and alternative oncogenic pathways were activated through MDM2 upregulation and p53 inactivation. To our surprise, the authors also exhibited an unexpected discrepancy between in vitro and in vivo treatments (oncogene inactivation in combination with autophagy-targeted senolysis), which as they support highlights the complexity of translating promising in vitro results to in vivo settings.
Although their working hypothesis is interesting, it is not sufficiently supported by their findings. The text is not easy to follow in some parts of the text especially those describing experimental procedures/manipulations which are complicated and should be better and in more detail described. Moreover, the validity of the in vitro findings related to treatment applied in vivo is absent, raising concerns.

Sprcific comments
• The authors mention that oncogene-inactivation senescence has been also described for other than Tag oncogenes such as Myc in lymphoma cells but they should expand the analysis in their experimental settings with an additional oncogene, in order to test whether this is a specific or more global phenomenon.
• The idea that genomic instability leads to senescence that is followed by reshuffling of the genomic landscape and activation of other oncogenic routes, driving escape from and tumor relapse has been extensively presented in the literature and is not new. The same is also valid for p53 inactivation and polyploidy as a mechanism of escape from oncogene induced senescence. Moreover, the senescence phenotype has been linked with stemness, resulting in aggressive behavior of escaped cells and tumor recurrences. Overall, the above raise issues regarding the novelty of the findings.
• The authors should provide more mechanistic insights regarding the oncogene-inactivation senescence phenotype, presented in the study.
• It is not satisfactory explained by the authors why in vitro findings in terms of autophagy inhibition are not found valid in vivo.

Minor comments
Results, Line 141-142: Given that senescence is an important aspect of the current study, the senescence phenotype upon

oncogene inactivation, should be verified by co-staining of cells with SA-b-Gal and other senescence markers.
Results, Line 179-181: Gene set enrichment analysis revealed pathways with differential expression between senescent and non-senescent cells. Given that p53 signaling pathway is one of the pivotal pathways that are differentially expressed between senescent and non-senescent cells, the reason why this is not identified in this analysis should be explained.
Results, Line 205-240: This section should be improved as it is not easy to follow and conceptually clear. A graphical figure presenting the workflow of the experiments performed would be beneficial towards this direction.

Reviewer #3

(Remarks to the Author)
This manuscript from Blankenstein's group investigated mechanisms of oncogene inactivation-induced senescence in tumorigenesis. Authors found morphological and metabolic changes in senescent cells. Autophagic activation might be associated with cell death of senescent cells. Cancer cell senescence is an important topic for overcoming therapeutic limitations and better treatment strategies for patients with cancer. However, the significance of the main findings is limited. Also, the cell lines are very specialized and were derived from one tumor, fibroblast originated, therefore, the biological correlation between the findings and cancer development/senescence in patients is not strong.
Below are major comments regarding this manuscript.
1. It is not clear whether this study focused on a certain cancer type, such as gastric cancer or sarcoma, or the general biology of cancer cells. The cell lines used for the studies are 1) clone 4 cells, characterized as gastric carcinoma, and 2) TTC#3055 characterized as spindle cell sarcoma. However, the origin cells of both lines seem to be fibroblast and it is not clear whether this is relevant to human cancer development and cancer origin.
2. Since cancer cell senescence can be regulated by both intrinsic and extrinsic factors, it would be important to examine the microenvironment in the tumors treated with or without doxycycline and address whether the extrinsic factors did not impact the cancer cell senescence at all.
3. I wonder whether the doxycycline concentration (1mg/ml) for in vitro culture is too high. Doxycycline can control cytokine secretion and is considered an anti-inflammatory drug when the concentration is high and the same concentration used for in vitro studies was used for in vivo studies. Thus, I wonder whether the anti-inflammatory signatures, such as IL6, and MMP secretion in senescent cells are from the doxycycline effect.
4. It is not clear whether the glycolysis pathway was reprogrammed into another metabolism after the cancer cells entered the senescence. Although there is a change in the glycolysis pathway, it is known that metabolism remains active in senescent cells. So, I wonder whether the changes in the glycolytic rate occur for senescent cell survival. More evidence to support the metabolic reprogramming should be provided. Should the data be interpreted as a metabolic response, not reprogramming?
5. The change in Cdkn1a gene expression is only about 1.5 fold. It needs to be confirmed whether the change in protein expression is more dramatic if the Cdkn1a is associated with the cell cycle arrest phenotype in senescent cells.
6. Is the histology of tumors before and after entering the senescence maintained although the tumor size is smaller? Are the features observed in vitro in senescent cells, such as flattened and irregularly shaped phenotypes, observed in tumors 7 or 12 days after the dox treatment in Figure 3B? Also, I wonder whether the histology of tumors from clone 4 cells is carcinoma or changed to another type.
7. If the senescent and active states are reversible, are the senescent cell features not observed in the re-isolated cell lines from relapsed tumors? I also wonder how the metabolic signatures were altered in the re-isolated cell lines.

Reviewer #4

(Remarks to the Author)
I co-reviewed this manuscript with one of the reviewers who provided the listed reports. This is part of the Nature Communications initiative to facilitate training in peer review and to provide appropriate recognition for Early Career Researchers who co-review manuscripts.

Version 1:

Reviewer comments:

Reviewer #1

(Remarks to the Author)

The authors of the manuscript by Schmitt et al. have provided a revised version of the manuscript that addresses a number of the concerns raised throughout the initial review process. However, not all of the concerns were sufficiently addressed, and some new issues have arisen. We outline below how these issues can be addressed:
• The reviewers do include data in human BRAFV600E-mutant melanoma cells showing that pharmacologic BRAF inhibition with vemurafenib induces hallmark features of senescence, which supports the proposition of OIIS in another model. However, (i) the authors do not show whether OIIS induced by vemurafenib is also transient (which is the main finding of this work), and (ii) unexpectedly introduce this data later in the manuscript instead of being supportive of their model in Figure 1/Extended Figure 1.
• We found it difficult to follow the analysis of the data presented in Figure 3 and extended Figure 3. The authors are requested to provide a more detailed explanation of the differences between transient and persistent senescent tumor cells

as defined by these experiments. What is the difference between the data for total flux and proliferating cells in Figures 3E and 3F? Precisely how/why are the “proliferating cells” suppressed in the CM2 model mice? If the senescence is persistent in Figure 3G, why/how are these tumor cells recovering? Why/how are the tumors being eliminated in Extended Figures 3B and 3E. These are central and critical experiments that do not receive sufficient explanation in the text.
• The authors make an argument for the importance of p53 and mdm2 in relapsed tumors but do not address how the current work might relate to the preponderance of tumors where p53 is mutant or null, despite the previous concern of improving the discussion of the role(s) of p53 and mdm2.
• In Figure 5B and extended Figure 5A, is the decline in flux and tumor size, respectively, a reflection of cell death? Is there any data to indicate precisely what is occurring in these tumors?
• The data relating to autophagy and autophagy inhibition in Figures 6 and Extended Figure 6 are not sufficiently rigorous to make any argument as to the involvement (or lack thereof) of autophagy in the observed outcomes. The extent of apoptosis (cell death) upon inclusion of Lys05 in Figure 6F is modest, at best. Only a single autophagy inhibitor is tested here and without genetic silencing of autophagy. Beclin is silenced in the experiments in Extended Figure 6, but Beclin-independent autophagy is well established. Silencing of other ATG/autophagy genes might have generated more convincing and rigorous outcomes. In Extended Figure 6C, it is unclear why these studies were not performed for the + vemurafenib conditions. The LC3 Western blots are not particularly convincing and were not quantified with statistics. The same concern applies to the pro-caspase 3 blots. Finally, there are any number of reasons why there was no impact of Lys05 in vivo in Figures 6H, 6I and Extended 6I, J, L and M, most prominently that the drug never achieved concentrations sufficient to inhibit autophagy in the tumor cells. There is no evidence presented that autophagy has actually been inhibited, and other pharmacological and genetic inhibitors of autophagy could/should have been examined. In case the authors are unable to adequately address these questions, the primary suggestion would be to eliminate the data pertinent to the role of autophagy in this system.

Reviewer #2

(Remarks to the Author)
While the authors have put sufficient efforts to address many of my previous issues, there are still serious concerns related to the scientific content and the concept of the revised work that have not been satisfactorily dealt with. Specifically:
Precise documentation of cellular senescence induction (and its inhibition) which is a milestone of the study, remains insufficient and unaddressed. It is well known that the SA-β-gal staining alone is not adequate to verify the senescence phenotype (Gorgoulis et al, Cell 2019; Ogrodnik et al, Cell 2024). Similarly, complementing SA-β-gal staining with the expression levels of senescence-related but not specific factors such as p21 or p16 in “bulk” material is a nonacceptable approach and can lead to false conclusions. p21 is expressed in transiently arrested cells or in non-senescent cells exhibiting DNA damage signaling (Karimian et al, DNA Repair 2016). Moreover, p21 is not universally expressed by senescent cells (Michaloglou et al, Nature 2005; Gray-Schopfer et al Br J Cancer 2006). Similarly, p16 has been reported to be expressed in a variety of non-senescence contexts (Safwan-Zaiter et al, Life 2022). Regarding SASP factors, they can be involved in a variety of other non-senescence contexts such as inflammatory or immune activation responses, leading to cross-reactivity and false-positive results (Gorgoulis et al, Cell 2019; Ogrodnik et al, Cell 2024). All the above motivated the scientific community to propose a guideline algorithm for accurate senescence detection (Gorgoulis et al, Cell 2019, Kohli et al, Nat Protoc 2021). The authors should clearly show the induction of cellular senescence (and its inactivation) in their cellular settings by applying co-stainings with established senescence markers according to this algorithm. In this manner, they will prove that the senescence phenotype (and its inactivation) presented in the current work is true. In line with the above, implementation of the Fridman signature although helpful is not sufficient, as it presents some flaws related to its specificity (Ntintas et al, FEBS Bio 2025). The signature was derived from literature-based meta-analysis rather than high-throughput omics data, and many of the original reference studies relied on indirect or non-specific senescence markers, including oxidative stress genes and cell cycle regulators, whose interpretation is now considered not specific to define true senescence (Fridman and Tainsky, Oncogene 2008).
In continuation to the previous, the authors state in lines 261-262 that “relapsed cells acquire a hybrid state, maintaining residual senescence features, while engaging proliferation and growth signalling programs”. This enhances the above concerns whether the phenotype identified by the authors as cellular senescence is indeed a true and full senescence program and not a transient and irrelevant state (for instance an outcome of oncogene addiction that can be related to growth arrest). Cells that escape from senescence are distinct from their paternal (senescent) ones acquiring new properties (altered genome and epigenome, increased invasion, resistance to anticancer agents and others) that have been related to cancer relapse (Milanovic et al Nature 2018; Galanos et al, NCB 2016; Zampetidis et al, Mol Cel 2021).
The authors provide some potential explanations on why the autophagy-targeted senolytic therapy did not turn out effective in vivo. This issue though remains a fundamental flaw of the current work, jeopardising its importance for developing new therapeutic avenues.
For the above reasons and given the calibre of the Journal in my opinion the current manuscript cannot be considered for publication in Nature Communications.

Reviewer #3

(Remarks to the Author)
I think that the authors have adequately addressed the comments and concerns raised by the reviewers in the revised version. Thus, I have no further comments.

Version 2:

Reviewer comments:

Reviewer #1

(Remarks to the Author)
The authors have conscientiously revised their manuscript in accord with the concerns raised in the original review.

Open Access This Peer Review File is licensed under a Creative Commons Attribution 4.0 International License, which permits use, sharing, adaptation, distribution and reproduction in any medium or format, as long as you give appropriate credit to the original author(s) and the source, provide a link to the Creative Commons license, and indicate if changes were made.
In cases where reviewers are anonymous, credit should be given to 'Anonymous Referee' and the source.
The images or other third party material in this Peer Review File are included in the article's Creative Commons license, unless indicated otherwise in a credit line to the material. If material is not included in the article's Creative Commons license and your intended use is not permitted by statutory regulation or exceeds the permitted use, you will need to obtain permission directly from the copyright holder.
To view a copy of this license, visit https://creativecommons.org/licenses/by/4.0/

Reviewer 1:

This manuscript by Schmitt and colleagues has the potential to contribute to the literature relating to senescence and escape from senescence. The strengths of this work are the sophisticated experimental approaches utilized, the relative novelty of studies relating to senescence induced by oncogene inactivation rather than the more well-established Oncogene-induced Senescence (OIS), and the findings relating to the regulation of this form of senescence and escape from senescence. In addition, the findings that oncogene-inactivation is transient and can facilitate long-term relapse, and that oncogene-inactivated senescent cells are amenable to senolysis by autophagy inhibition in vitro (but not in vivo) may represent important contributions to the field. However, the work has a number of deficiencies. One is that oncogene-induced senescence and its relationship to the current work is entirely ignored. There is also insufficient discussion of other studies in the literature involving senescence and escape from senescence both in tumor bearing animals and disease recurrence in patients such as the seminal work of Duy et al. and Milanovic et al. There is also some concern that the findings are based on a single, specific, genetically-engineered model.

We thank the reviewer for the constructive comments and fully agree that placing our work in the context of prior studies on OIS and senescence escape strengthens its impact. In the revised manuscript, we now explicitly differentiate oncogene-induced senescence (OIS) from oncogene inactivation-induced senescence (OIIS) and clarify how our findings extend the current understanding of therapy-induced relapse mechanisms. We have also incorporated references to key studies on therapy-induced senescence (TIS), including those by Duy et al. and Milanovic et al., which demonstrated that chemotherapy- or radiotherapy-induced senescence can be reversible and contribute to disease recurrence. While these studies focused on TIS, our work shows that a related phenomenon occurs after targeted oncogene inactivation, where OIIS similarly predisposes to tumor relapse, highlighting how senescence escape is a general principle across multiple therapeutic settings. Finally, we would like to clarify that our conclusions are not based on a single model: we used two independent murine tumor cell lines of distinct histological origin (epithelial and mesenchymal) and now include new validation data in human BRAFV600E-mutant melanoma cells, thereby further supporting the robustness and translational relevance of our findings.

1. The authors use the dox-switchable on-off Tag oncogene tumor cell model in their analyses. Chronic dox treatment results in the activation of Tag, which then drives cellular hyperproliferation consistent with malignant transformation. This is often associated with the development of "oncogene addiction" which can lead to growth arrest once "inactivated". This is similar to previously reported findings where the inactivation of an oncogene in oncogene- addicted cells can in fact lead to senescence: c-myc (PMID: 37656618; Afifi MM et al,. Irreversible cell cycle exit associated with senescence is mediated by constitutive MYC degradation) or HPV E7 (PMID: 31693922 Celegato M et al, A novel small-molecule inhibitor of the human papillomavirus E6-p53 interaction that reactivates p53 function and blocks cancer cells growth. Cancer Lett. 2020 Feb 1;470:115-125.) and PMID: 31603527). These previous findings must be considered in the formulation of a more in-depth interpretation of the data.

We have now incorporated and discussed the relevant work on c-MYC and HPV oncogene inactivation in the revised Discussion. These findings reinforce the broader

concept that oncogene withdrawal can trigger senescence. Our work builds on this principle by demonstrating that, in addition to inducing a senescent state, oncogene inactivation predisposes tumors to relapse through genomic instability, activation of alternative oncogenic pathways, metabolic adaptation, and remodeling of the tumor microenvironment. Thus, our study extends earlier observations by providing mechanistic insight into how oncogene inactivation–induced senescence not only constrains tumor growth but also creates conditions that favor disease recurrence.

2. The dox-switchable on-off Tag oncogene tumor cell model allows for promotion or inhibition of the expression of Tag rather than activation/deactivation of the oncogene. The only way to confirm the primary proposal in this manuscript (i.e. that oncogene inactivation rather than activation is what drives senescence) is to directly (pharmacologically or genetically) inactivate oncogenes in various tumor cell models (such as RAS, B-Raf, and c-myc, among others). Inactivating one of these oncogenes in another cellular model would provide proof-of-concept and support the central proposition of the manuscript.

We would like to clarify that in our system SV40 large T antigen (Tag) functions as the oncogenic driver, and the doxycycline switch enables its regulated expression. Thus, Tag inactivation in our model directly represents oncogene inactivation. Importantly, our intention is not to claim that oncogene inactivation per se, rather than activation, drives senescence. Instead, our work identifies oncogene inactivation–induced senescence (OIIS) as an additional mode of senescence induction that is particularly relevant for residual disease in cancers treated with targeted oncogene inhibitors. To further strengthen this point, we have now included data in human $BRAF^{V600E}$-mutant melanoma cells, showing that pharmacologic BRAF inhibition with vemurafenib induces hallmark features of senescence and confers partial senolytic sensitivity. These results demonstrate that OIIS is not confined to the Tag model and extend its relevance to human cancer models. Finally, in response to the reviewer's concern, we have revised and expanded both the Introduction and Discussion to provide a more balanced and contextualized view of different oncogenes (including c-MYC, BRAF, and KRAS) whose inactivation has been shown to induce senescence.

3. While the acute reduction in Tag expression clearly induces the expression of p21, this is also accompanied by p16 downregulation, which is unusual. The authors attempt to explain this observation by the assumption that Tag withdrawal-induced senescence is dependent on p21 and not p16; however, this hypothesis requires further experimental evaluation (e.g., knocking down either protein and observing the outcome on senescence induction in vitro).

We agree with the reviewer that the observed downregulation of p16 upon Tag withdrawal is unusual. This finding has been confirmed by western blot and is now presented in the revised manuscript. Importantly, this phenomenon is not unprecedented and has been described in earlier Tag-driven models, where restoration of Rb function following Tag inactivation represses p16 expression. In line with this, our Discussion has been expanded to provide mechanistic context based on the regulatory interplay between Tag, Rb, and p16. Moreover, we note that p16 was also not upregulated in our human BRAFV600E melanoma model upon oncogene inactivation despite cells showing clear features of senescence. This parallel finding supports the idea that noncanonical, p21-dependent but p16-independent senescence programs can arise in distinct contexts.

Figure 1: This figure absolutely requires quantification of senescence, which is the fundamental observation underlying all subsequent studies. Images of beta galactosidase staining in the cells are insufficient. It is further insufficient to only measure expression (message levels) of p21 and p16; data must be provided relating to protein levels, as is consistent throughout the literature. Finally, the authors refer to Rb as a critical factor relating to senescence induction, but neglect to assess levels of phosphorylated (inactive) and dephosphorylated Rb (the active form that is considered to be critical for promotion of senescence as well as levels of the Rb-E2F complex(es), where binding to the dephosphorylated form of Rb by E2F arrests the cell.

All SA-β-Gal images were quantified, and the ratio of positive to negative cells was calculated from three independent experiments. Both p21 and p16 have been assessed at the protein level by western blot, and these data are now included in the revised manuscript. With respect to Rb, the direct interaction between Tag and Rb complicates the interpretation of phosphorylation dynamics in our mouse model, as Tag binding inactivates Rb by preventing its interaction with E2F. In clone 4 cells, we observed a transient increase in phosphorylated Rb at 6 hours after doxycycline withdrawal, followed by a decline, but these changes are difficult to interpret in light of Tag–Rb binding. For this reason, we did not include the data in the main manuscript. Importantly, however, we consistently observed upregulation of p21 upon Tag withdrawal, which is consistent with the induction of senescence (Figure 1G).

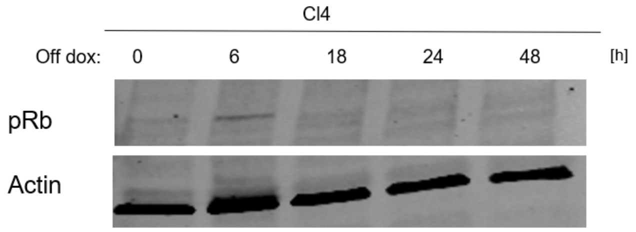

In contrast, in A375 BRAF^{V600E} melanoma cells, where Tag–Rb interactions are not present, we observed a marked downregulation of phosphorylated Rb upon vemurafenib treatment (new Extended Figure 6A). In agreement with the reviewer's point, this reduction in pRb is consistent with the promotion of senescence that we observe in this human model.

Figure 2. Were these data generated only from a single clone? This is insufficient to allow the authors or readers to draw conclusions relating to the broader relevance of these observations. The data must be confirmed by studies in another model of oncogene inactivation induced senescence. Moreover, transcriptomic analysis must consider the application of well-established senescence signatures such as Fridman's UP (which provides a comprehensive transcriptomic signature of gene expression and pathways indicative of senescence).

The transcriptomic data in Figure 2 were generated from biological replicates derived from independent cultures of the same clone (clone 4). While this transcriptomic analysis was performed in one cell line, the senescent phenotype was validated across multiple independent clones and in an additional murine cancer cell line (TTC#3055), as well as in human BRAF^{V600E}-mutant melanoma cells, strengthening the robustness and generalizability of our observations. As suggested, we have now included in the

revised manuscript an analysis of established senescence gene signatures, including Fridman's SENESCENCE_UP, which shows strong enrichment in our dataset.

Figure 3. The conclusions relating to mdm2 and p53 are intriguing. Unfortunately, the authors have neglected to demonstrate that the pharmacological mdm2 inhibitor utilized in these studies has a direct impact on mdm2 and p53. Moreover, despite being state-of-the-art, studies in Figure 3 only provide proof that "transient" or "escapable" senescence can predispose to cancer relapse rather than providing a specific interpretation of this phenomenon with respect to oncogene-inactivation. These data are important and relevant to the proposal that tumor cell senescence can serve as a mechanism for cancer relapse, but only if more thoughtfully interpreted. Findings related to mdm2 dependence must be discussed in the light of PMID: 29402901 (Small molecule antagonists attenuate the senescence associated secretory phenotype).

We appreciate the reviewer's point. We recognize that biochemical validation of the MDM2–p53 axis following AMG-232 treatment (e.g., demonstration of reduced MDM2 activity and increased p53 stabilization) is the standard approach. In our study, we focused on the functional dependency of relapsed cells on MDM2 and therefore did not repeat these biochemical assays, which have already been comprehensively established for AMG-232 in prior studies. Our data show that relapsed, but not parental, cells are selectively sensitive to AMG-232, thereby functionally confirming MDM2 dependence. To strengthen the broader interpretation, we have expanded our analysis on the effects of OIIS in vivo in the revised manuscript. In particular, we now provide a detailed analysis of the remodeling of the tumor microenvironment using spectral flow cytometry and UMAP-based clustering, which captures dynamic changes across tumor progression, remission, and relapse. This analysis reveals shifts in immune and stromal compartments in the context of oncogene inactivation–induced senescence and its effects in facilitating relapse. Finally, we explicitly discuss our MDM2-related findings in the context of the suggested literature, highlighting the dual role of MDM2 inhibitors in both modulating p53 activity and attenuating SASP.

Figure 5. The Y axis in Figure 5A is indicated to demonstrate "relative cell death". This is an inappropriate measurement and instead the authors must indicate the actual percentage of cells that die, as is the case for the apoptosis measurements. With respect to autophagy, which is a central component of the latter portion of the manuscript, the one assay that is utilized (LC3I and II levels) does not provide convincing data. Additional assays evaluating autophagy induction and suppression with both pharmacological and genetic autophagy inhibition must be provided to support any conclusions relating to the relationship between autophagy and senescence in these studies.

We have included the actual percentage of dead cells in Figure 5A, replacing the previous representation of relative cell death. With respect to autophagy, our results include both pharmacological (Lys05) and genetic inhibition of autophagy (Beclin-1 knockdown). These experiments consistently show that autophagy suppression sensitizes senescent cells to cell death in vitro, thereby strengthening our conclusion that senescent cells depend on autophagy for survival under oncogene-deprived conditions. In addition, we extended these analyses to human $BRAF^{V600E}$ melanoma cells, where vemurafenib-treated A375 cells showed increased sensitivity to Lys05. This finding confirms that the autophagy dependence of senescent cells is not restricted to the Tag model but is also observed in a clinically relevant human system.

Figure 5. The results in the in vivo experiment presented in Figure 5E are striking in largely contradicting one of the primary concepts discussed in the latter section of manuscript, specifically that inhibition of autophagy would cull oncogene inactivation-induced senescence. An alternative explanation is that autophagy is likely to facilitate an immune response against senescent cells (or perhaps just tumor cells) which results in the observed contradictory effect in immunodeficient mice. In an effort to extend and confirm this observation, tumor-bearing animal studies relating to autophagy must also be provided utilizing cells where Beclin has been suppressed (as in the cell culture work).

As correctly noted, our in vivo findings in immunocompetent mice contrast with the senolytic effects of autophagy inhibition observed in vitro. We agree with the reviewer that autophagy may play a dual role, not only supporting the survival of senescent tumor cells but also facilitating immune responses against them. This possibility is now explicitly acknowledged and discussed in the revised manuscript. Our spectral flow cytometry analyses demonstrate substantial remodeling of the tumor microenvironment across progression, remission, and relapse, consistent with the idea that immune context influences therapeutic outcome. We also highlight that this may explain the difference between our mouse model and the A375 xenograft experiment, where Lys05 treatment produced a modest delay in tumor growth. In the immunocompetent setting, autophagy blockade could impair immune cell function. This interpretation has been incorporated into the revised Discussion. While we agree that in vivo studies using Beclin-1 knockdown cells would provide further mechanistic dissection of cell-intrinsic versus immune-mediated roles of autophagy, we respectfully note that such experiments extend beyond the scope of the present study. We consider this an important and promising avenue for future work.

Reviewer 2

[…] The authors mention that oncogene-inactivation senescence has been also described for other than Tag oncogenes such as Myc in lymphoma cells but they should expand the analysis in their experimental settings with an additional oncogene, in order to test whether this is a specific or more global phenomenon.

We thank the reviewer for this helpful suggestion. In the revised manuscript, we now include data from human BRAFV600E-mutant melanoma cells, where pharmacologic mutant BRAF inhibition with vemurafenib induces hallmark features of OIIS, including senescence markers and partial senolytic sensitivity. These findings confirm that OIIS is not limited to the Tag system and support its broader relevance to other oncogenes targeted in human cancer therapy. While a comprehensive analysis of multiple additional oncogenes lies beyond the scope of this study, the inclusion of the BRAF model provides proof-of-concept that oncogene inactivation–induced senescence is a more generalizable phenomenon.

The idea that genomic instability leads to senescence that is followed by reshuffling of the genomic landscape and activation of other oncogenic routes, driving escape from and tumor relapse has been extensively presented in the literature and is not new. The same is also valid for p53 inactivation and polyploidy as a mechanism of escape from oncogene induced senescence. Moreover, the senescence phenotype has been linked

with stemness, resulting in aggressive behavior of escaped cells and tumor recurrences. Overall, the above raise issues regarding the novelty of the findings.

We fully agree that genomic instability, p53 inactivation, polyploidy, and links between senescence and stemness have been described in the context of oncogene-induced senescence (OIS) and therapy-induced senescence (TIS). The novelty of our study lies in demonstrating that these processes also occur in the setting of oncogene inactivation–induced senescence (OIIS), a distinct and previously underexplored form of senescence relevant to targeted therapy. By providing functional, metabolic, and immunological characterization of OIIS, and validating key aspects in a human BRAF-mutant model, we extend the senescence paradigm beyond OIS and TIS, establishing OIIS as an additional mechanism that primes tumors for relapse.

The authors should provide more mechanistic insights regarding the oncogene-inactivation senescence phenotype, presented in the study.

In the revised manuscript, we have expanded the mechanistic analysis of the OIIS phenotype to include both cell-intrinsic and non–cell-autonomous components. At the cell-intrinsic level, we now detail the contributions of polyploidy, Mdm2 upregulation, and metabolic adaptations to senescence maintenance and escape. At the non–cell-autonomous level, we present a comprehensive characterization of stromal and immune remodeling using spectral flow cytometry and UMAP-based profiling across progression, remission, and relapse. Together, these additions provide a more mechanistic framework for understanding how oncogene inactivation–induced senescence both constrains tumor growth and predisposes to relapse.

It is not satisfactory explained by the authors why in vitro findings in terms of autophagy inhibition are not found valid in vivo.

We agree that this is an important point. We believe the discrepancy between the in vitro and in vivo results arises from fundamental differences in the metabolic and microenvironmental context. In vitro, senescent cells rely heavily on autophagy for survival, and its inhibition is strongly senolytic. In vivo, however, senescent cells are embedded in a remodeled tumor microenvironment, where stromal and immune interactions can provide compensatory survival cues that attenuate the effect of autophagy blockade. This is exemplified by the difference between our two models: in A375 xenografts grown in immunodeficient mice, we observed a modest delay in tumor growth with Lys05, whereas in clone 4 tumors in immunocompetent mice, Lys05 tended to accelerate relapse. We now discuss that this divergence could reflect the impact of autophagy inhibition on immune function, particularly T cells, in the immunocompetent setting, which may counteract senolytic effects. In the revised manuscript, we have expanded the Discussion accordingly. While further mechanistic dissection is beyond the scope of the present study, these findings highlight the importance of immune context and identify promising directions for future research.

Results, Line 141-142: Given that senescence is an important aspect of the current study, the senescence phenotype upon oncogene inactivation, should be verified by co-staining of cells with SA-b-Gal and other senescence markers.

As an additional senescence marker, we stained senescent cells using an antibody specific for uPAR; however, the results were not clear or consistent, and we note that

uPAR has not emerged as a universal marker across different senescence-inducing models in our experience. For this reason, we did not include uPAR-based analyses in the revised manuscript. Instead, we provide multiple lines of evidence that together robustly establish the senescence phenotype. In addition to SA-β-Gal staining and p21 protein upregulation, our transcriptomic analyses reveal significant enrichment of senescence-associated gene sets (e.g., FRIDMAN_SENESCENCE_UP) in oncogene-inactivated cells, as well as SASP components including Mmp3, and Il6. We believe these combined morphological, biochemical, and transcriptomic data provide a comprehensive validation of the senescence phenotype in our models.

Gene set enrichment analysis revealed pathways with differential expression between senescent and non-senescent cells. Given that p53 signaling pathway is one of the pivotal pathways that are differentially expressed between senescent and non-senescent cells, the reason why this is not identified in this analysis should be explained.

In the original analysis, the global pathway enrichment did not highlight “p53 signaling pathway” among the top hits, which may have created the impression that p53 activity was absent in OIIS. In the revised version of the manuscript, we have re-analyzed the transcriptomic data using complementary gene set collections and more stringent thresholds. This revealed that while overall p53 transcript levels decrease upon Tag withdrawal, there is in fact selective enrichment of p53 target gene signatures. Specifically, our GSEA with the ONGUASAHA_TP53_TARGETS gene set shows that senescent cells upregulate a subset of p53-responsive genes. This finding is consistent with our observation that Tag withdrawal destabilizes p53, leading to lower overall expression, but that residual p53 activity is sufficient to drive senescence through p21 induction. We now include these data explicitly in the Results and have revised the Discussion to clarify that OIIS represents a “p53-low, p21-dependent, p16-independent” state, supported by transcriptomic evidence of enriched p53 target gene activity despite reduced p53 levels.

Results, Line 205-240: This section should be improved as it is not easy to follow and conceptually clear. A graphical figure presenting the workflow of the experiments performed would be beneficial towards this direction.

We fully acknowledge that the experimental system is complex and that this section of the Results required clearer presentation. In the revised manuscript, we have substantially improved the explanation and added new graphical schemes summarizing the workflow of the animal experiments. These figures are designed to provide a step-by-step overview of the experimental setup, treatment schedules, and readouts, thereby making the Results easier to follow and conceptually clearer. We hope these additions address the reviewer’s concern and improve the accessibility of this section.

Reviewer 3

This manuscript from Blankenstein’s group investigated mechanisms of oncogene inactivation-induced senescence in tumorigenesis. Authors found morphological and metabolic changes in senescent cells. Autophagic activation might be associated with cell death of senescent cells. Cancer cell senescence is an important topic for overcoming therapeutic limitations and better treatment strategies for patients with

cancer. However, the significance of the main findings is limited. Also, the cell lines are very specialized and were derived from one tumor, fibroblast originated, therefore, the biological correlation between the findings and cancer development/senescence in patients is not strong.

The cancer cell lines used in this study were derived from tumors arising in different mice and represent distinct tumor entities (epithelial and mesenchymal origin). We believe these models closely resemble human tumors displaying oncogene addiction and are therefore well suited to study oncogene inactivation-induced senescence and cancer relapse. In the revised manuscript, we have further strengthened the translational relevance by including data from human BRAF^{V600E}-mutant melanoma cells, which reproduce key hallmarks of OIIS, underscoring that our findings extend beyond the original murine models.

1. It is not clear whether this study focused on a certain cancer type, such as gastric cancer or sarcoma, or the general biology of cancer cells. The cell lines used for the studies are 1) clone 4 cells, characterized as gastric carcinoma, and 2) TTC#3055 characterized as spindle cell sarcoma. However, the origin cells of both lines seem to be fibroblast and it is not clear whether this is relevant to human cancer development and cancer origin.

TTC#3055 cells have a mesenchymal origin (spindle cell sarcoma), while clone 4 cells have an epithelial origin (gastric carcinoma), supporting the view that our findings are relevant to different tumor types. We have clarified this point in the revised manuscript. Furthermore, we have strengthened the translational relevance by including human BRAF$_{V600E}$-mutant melanoma cells, in which key features of OIIS were reproduced. Our overarching concept is that OIIS represents a generalizable mechanism, potentially applicable across diverse cancer types.

2. Since cancer cell senescence can be regulated by both intrinsic and extrinsic factors, it would be important to examine the microenvironment in the tumors treated with or without doxycycline and address whether the extrinsic factors did not impact the cancer cell senescence at all.

In the revised manuscript, we have addressed this by performing a detailed analysis of the tumor microenvironment during OIIS using spectral flow cytometry and UMAP-based clustering. This analysis revealed pronounced stromal remodeling upon doxycycline withdrawal, including immune cell shifts. These findings indicate that extrinsic factors are indeed altered during OIIS and likely contribute to the biology of senescence and relapse in vivo.

3. I wonder whether the doxycycline concentration (1mg/ml) for in vitro culture is too high. Doxycycline can control cytokine secretion and is considered an anti-inflammatory drug when the concentration is high and the same concentration used for in vitro studies was used for in vivo studies. Thus, I wonder whether the anti-inflammatory signatures, such as IL6, and MMP secretion in senescent cells are from the doxycycline effect.

In our system, senescence is induced by withdrawal of doxycycline rather than its presence. Importantly, we observe increased secretion of IL-6 and Mmp3 when cells are taken off doxycycline, concomitant with senescence induction. Thus, the SASP

phenotype, including IL-6 upregulation, arises specifically under doxycycline withdrawal conditions. In this context, we do not believe that doxycycline interferes with the induction of senescence or accounts for the pro-inflammatory signatures we report. Moreover, since IL-6 is considered primarily pro-inflammatory within the SASP, the presence of doxycycline is unlikely to confound the inflammatory phenotype we describe.

4. It is not clear whether the glycolysis pathway was reprogrammed into another metabolism after the cancer cells entered the senescence. Although there is a change in the glycolysis pathway, it is known that metabolism remains active in senescent cells. So, I wonder whether the changes in the glycolytic rate occur for senescent cell survival. More evidence to support the metabolic reprogramming should be provided. Should the data be interpreted as a metabolic response, not reprogramming?

To test whether changes in the glycolytic rate occur for senescent cell survival, as suggested by the reviewer, we tested the sensitivity of senescent cells to 2-DG (new Extended Figure 2G). Indeed, we observed that senescent cells were highly sensitive to glycolysis inhibition, showing a marked increase in cell death compared to proliferating cells. Similar results were observed when we treated senescent cells with mitochondrial inhibitors. This demonstrates that senescent cells rely on glycolysis and mitochondrial function for survival. In line with the reviewer's comment, we also rephrased our interpretation to describe this phenotype as a metabolic adaptation supporting senescent cell survival, rather than a complete metabolic reprogramming.

5. The change in Cdkn1a gene expression is only about 1.5 fold. It needs to be confirmed whether the change in protein expression is more dramatic if the Cdkn1a is associated with the cell cycle arrest phenotype in senescent cells.

To address this point, we assessed p21 expression at the protein level by Western blot. As shown in the revised manuscript (new Figure 1G), we observed a clear increase in p21 protein expression upon doxycycline withdrawal, consistent with its role in mediating cell cycle arrest in senescent cells.

6. Is the histology of tumors before and after entering the senescence maintained although the tumor size is smaller? Are the features observed in vitro in senescent cells, such as flattened and irregularly shaped phenotypes, observed in tumors 7 or 12 days after the dox treatment in Figure 3B? Also, I wonder whether the histology of tumors from clone 4 cells is carcinoma or changed to another type.

We attempted histological analyses, but at early remission stages (7–12 days after doxycycline), residual Matrigel interfered with SV40 Tag staining, which is essential for assessing oncogene expression in our model. This technical limitation prevented reliable histological characterization at those time points. However, to obtain quantitative information on cellular composition, we opted for spectral flow cytometry instead. Consistent with the senescent morphology observed in vitro, our spectral flow cytometry analyses show that tumor cells display increased size during remission compared to progressive or relapsed tumors, in line with the enlarged and flattened morphology characteristic of senescent cells. Importantly, when senescent cells (off doxycycline) are re-exposed to doxycycline to re-induce oncogene expression and proliferation, they lose this enlarged phenotype, consistent with the forward and side scatter profiles observed. These findings indicate that key morphological features of

senescence are maintained in tumors following oncogene inactivation but are reversible once the oncogene is re-expressed and cells re-enter the cell cycle.

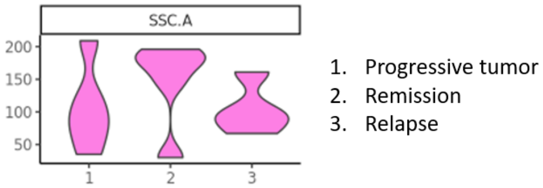

7. If the senescent and active states are reversible, are the senescent cell features not observed in the re-isolated cell lines from relapsed tumors? I also wonder how the metabolic signatures were altered in the re-isolated cell lines.

We thank the reviewer for this insightful comment. As the reviewer suggested, we have analyzed senescent and metabolic features of re-isolated cell lines derived from relapsed tumors. As now detailed in the Results section (Figures 4A–F, Extended Figures 4A–F, and Supplementary Document 2), relapsed cells still show features reminiscent of the previous senescent state but concurrently upregulate proliferative and growth factor signaling pathways (MAPK, PI3K–AKT, cytokine–receptor interactions). Metabolic pathway analysis revealed a shift away from glycosaminoglycan and sphingolipid metabolism toward anabolic pathways such as nucleotide, amino acid, and folate cycle metabolism, consistent with enhanced proliferative demands. Altogether, these additional analyses directly address the reviewer's concern and demonstrate that relapse after OIIS is accompanied by loss of senescent features, acquisition of proliferative and metabolic adaptations.

We are grateful to the Reviewers for their constructive criticism. Below please find a detailed point-by-point reply.

Reviewer #1:

The authors of the manuscript by Schmitt et al. have provided a revised version of the manuscript that addresses a number of the concerns raised throughout the initial review process. However, not all of the concerns were sufficiently addressed, and some new issues have arisen. We outline below how these issues can be addressed:

• The reviewers do include data in human BRAFV600E-mutant melanoma cells showing that pharmacologic BRAF inhibition with vemurafenib induces hallmark features of senescence, which supports the proposition of OIIS in another model. However, (i) the authors do not show whether OIIS induced by vemurafenib is also transient (which is the main finding of this work), and (ii) unexpectedly introduce this data later in the manuscript instead of being supportive of their model in Figure 1/Extended Figure 1.

We thank the reviewer for this important point. We agree that directly addressing the *transience* of oncogene inactivation-induced senescence (OIIS) in the BRAF-mutant melanoma model is crucial for the overall concept of the study. In the revised manuscript, we now provide both in vitro and in vivo evidence that vemurafenib-induced OIIS in A375 melanoma cells is not durably stable. First, we treated A375 cells with vemurafenib in vitro to induce growth arrest and acquisition of senescence markers. Upon withdrawal of vemurafenib, cells resumed proliferation (see Extended Data Fig. 3K). Second, to assess transience in vivo, we pretreated A375 cells with vemurafenib in vitro to induce senescence, implanted them subcutaneously, and maintained recipient mice on vemurafenib for four weeks. Despite extended vemurafenib treatment, tumors ultimately formed and grew (Extended Data Fig. 3M), consistent with a senescent state that is not durably maintained in this setting.

We further agree that the vemurafenib/A375 data are better placed early in the manuscript to support our model. In the revised version, we have therefore reorganized both the figures and the corresponding text.

• We found it difficult to follow the analysis of the data presented in Figure 3 and extended Figure 3. The authors are requested to provide a more detailed explanation of the differences between transient and persistent senescent tumor cells as defined by these experiments. What is the difference between the data for total flux and proliferating cells in Figures 3E and 3F? Precisely how/why are the “proliferating cells” suppressed in the CM2 model mice? If the senescence is persistent in Figure 3G, why/how are these tumor cells recovering? Why/how are the tumors being eliminated in Extended Figures 3B and 3E. These are central and critical experiments that do not receive sufficient explanation in the text.

1. Transient vs persistent senescent tumor cells (and “never senescent” controls)
We recognize that the term “persistent senescence” may have been confusing. To address this, we have revised the wording throughout the manuscript to “long-term senescence,” which more accurately reflects our observations and experimental design. “Long-term senescence” in our model refers to the experimental condition in which the senescent state is induced in vitro upon oncogene inactivation and maintained for an extended period when the cells are subsequently implanted in vivo under continuous oncogene inactivation. Upon injection of oncogene-inactivated senescent cells into immunocompetent mice, we observe that cells can eventually form tumors without dox-mediated reactivation of TagLuc. These relapsed tumors are not dependent on TagLuc and show increased genomic instability, which likely facilitates the acquisition of alternative oncogenic pathways. This supports our “transient senescence model,” in which acquisition of a senescent state predisposes cells to relapse at later stages.

In addition, the different experimental scenarios are now summarized in a new schematic Figure (Figure 3). The scheme illustrates for each experimental situation the dox regimen, the state of TagLuc expression, and whether cells are senescent or proliferating at each stage. We have added explanatory sentences to the Results text (p. 5 and 6 in the manuscript, marked yellow). Together with the new schematic Figure 3, these changes clarify how transient OIIS, long-term OIIS and never senescent are defined and used in the experiments.

2. Difference between total flux and proliferating cells (Extended Figures 3E and 3F)

We agree that the distinction between "total flux" and "proliferating tumors/cells" needed to be clearer. We have now clarified this in the Results text and figure legend:

- "Tumor burden was quantified by tumor volume and TagLuc-derived bioluminescent flux, the latter directly reporting the *in vivo* burden of oncogene-expressing tumor cells, as the firefly luciferase is fused to the oncogene Tag." (p. 5, marked yellow)

3. How/why proliferating cells are suppressed in CM2 mice

We appreciate the request for a more mechanistic explanation. We now explain more explicitly that tumor regression in CM2 mice is due to immune recognition of the Tag/SV40 oncogene once it is expressed. We added:

- "In CM2 mice, tumors initially grew but were consistently rejected by immune responses targeting TagLuc shown by staining of peptide IV-MHC I tetramer$^+$ CD8$^+$ T-cells (Figure 3A and Extended Figure 3A, B). Importantly, no tumor relapse was observed in these mice over a long observation period. In Rag2-KO mice, tumors grew progressively consistent with the absence of immune-mediated clearance (Figure 3A, Extended Figure 3E)." (p. 5, marked yellow)
- Thus, the proliferating cells are suppressed in CM2 mice because, once dox is present, they express TagLuc and become targets for an adaptive immune response that eliminates them.
- The mechanism is now also mentioned and specified in the newly added Figure 3.

• The authors make an argument for the importance of p53 and mdm2 in relapsed tumors but do not address how the current work might relate to the preponderance of tumors where p53 is mutant or null, despite the previous concern of improving the discussion of the role(s) of p53 and mdm2.

We agree that the relevance of our p53/Mdm2 findings for TP53-mutant or TP53-null tumors needed to be discussed more explicitly. To explicitly address this concern about the large fraction of tumors with TP53 alterations, we now clearly delimit the scope of our findings:

- "Nevertheless, our findings are most directly relevant to tumors that retain a functional p53 pathway but acquire Mdm2 overexpression or amplification, a genetic configuration present in several human malignancies." (p. 10, marked yellow)

This makes clear that we do not claim universal applicability across all tumor types, but specifically to p53-competent, Mdm2-driven contexts. We now directly discuss how OIIS and relapse might differ in TP53-mutant/null cancers, in line with the reviewer's request:

- "By contrast, in the many human cancers with TP53-mutations, OIIS and its escape are likely governed by p53-independent senescence programs and alternative forms of oncogene addiction, therefore Mdm2 inhibition would not be expected to provide the same therapeutic benefit." (p. 10, marked yellow)

This sentence explicitly states that, in TP53-mutant/null tumors, other pathways are expected to drive senescence escape, and Mdm2 inhibition is unlikely to have the same impact. Finally, we now frame the extension to TP53-mutant tumors as a question for future studies:

- "Whether analogous oncogene-addiction–like pathways are restored in relapses of TP53-mutant tumors, and whether such alternative senescence-escape circuits can likewise be leveraged therapeutically, will need to be addressed by future studies." (p. 10, marked yellow)

This directly addresses the raised concern by (i) acknowledging the limitation of our current model, (ii) avoiding overgeneralization, and (iii) explicitly pointing to the need for dedicated work in TP53-deficient settings.

• In Figure 5B and extended Figure 5A, is the decline in flux and tumor size, respectively, a reflection of cell death? Is there any data to indicate precisely what is occurring in these tumors?

We have clarified in the revised manuscript how bioluminescent flux and tumor size relate to each other. We now state more clearly that our interpretation of the flux and tumor volume decrease as reflecting tumor cell death is based on:

- the strong concordance of both readouts,
- the complete disappearance of palpable tumors at endpoint in CM2 mice, and
- the requirement for an intact adaptive immune system (no comparable decline in Rag2-KO mice).

In a previous study from our group, using a related Tag/TagLuc-driven tumor model (ref. 28, Anders, K. *et al. Cancer Cell* 2011) oncogene inactivation was shown to induce pronounced tumor cell death, as evidenced by histological analyses concomitant with tumor regression. This study is also cited in our manuscript. We hope that these clarifications, together with the expanded explanation of flux versus tumor size earlier in the Results, make our interpretation of the data in Figure 5B and Extended Figure 5A clearer.

• The data relating to autophagy and autophagy inhibition in Figures 6 and Extended Figure 6 are not sufficiently rigorous to make any argument as to the involvement (or lack thereof) of autophagy in the observed outcomes. The extent of apoptosis (cell death) upon inclusion of Lys05 in Figure 6F is modest, at best. Only a single autophagy inhibitor is tested here and without genetic silencing of autophagy. Beclin is silenced in the experiments in Extended Figure 6, but Beclin-independent autophagy is well established. Silencing of other ATG/autophagy genes might have generated more convincing and rigorous

outcomes. In Extended Figure 6C, it is unclear why these studies were not performed for the + vemurafenib conditions. The LC3 Western blots are not particularly convincing and were not quantified with statistics. The same concern applies to the pro-caspase 3 blots. Finally, there are any number of reasons why there was no impact of Lys05 in vivo in Figures 6H, 6I and Extended 6I, J, L and M, most prominently that the drug never achieved concentrations sufficient to inhibit autophagy in the tumor cells. There is no evidence presented that autophagy has actually been inhibited, and other pharmacological and genetic inhibitors of autophagy could/should have been examined. In case the authors are unable to adequately address these questions, the primary suggestion would be to eliminate the data pertinent to the role of autophagy in this system.

We agree with the reviewer that, as presented, our autophagy experiments might not be supportive of strong conclusions regarding the role of autophagy in OIIS escape or senolysis in our model. In light of these valid concerns, and to avoid overinterpreting our data, we have decided to remove the autophagy/senolytic part entirely from the revised manuscript as suggested by the reviewer. We consider this an interesting question for future, dedicated studies using more comprehensive genetic and pharmacological approaches, but no longer make claims in the present manuscript. We believe this change strengthens the manuscript by sharpening its focus on the central and best-supported aspect of our work: the characterization of OIIS and its genetic, metabolic, cytogenetic, and microenvironmental hallmarks. We now explicitly emphasize this conceptual focus in the revised Abstract and Discussion. We are grateful to this Reviewer for suggesting to delete this part from the manuscript.

Reviewer #2:

While the authors have put sufficient efforts to address many of my previous issues, there are still serious concerns related to the scientific content and the concept of the revised work that have not been satisfactorily dealt with.

Specifically: Precise documentation of cellular senescence induction (and its inhibition) which is a milestone of the study, remains insufficient and unaddressed. It is well known that the SA-β-gal staining alone is not adequate to verify the senescence phenotype (Gorgoulis et al, Cell 2019; Ogrodnik et al, Cell 2024). Similarly, complementing SA-β-gal staining with the expression levels of senescence-related but not specific factors such as p21 or p16 in "bulk" material is a nonacceptable approach and can lead to false conclusions. p21 is expressed in transiently arrested cells or in non-senescent cells exhibiting DNA damage signaling (Karimian et al, DNA Repair 2016). Moreover, p21 is not universally expressed by senescent cells (Michaloglou et al, Nature 2005; Gray-Schopfer et al Br J Cancer 2006). Similarly, p16 has been reported to be expressed in a variety of non-senescence contexts (Safwan-Zaiter et al, Life 2022). Regarding SASP factors, they can be involved in a variety of other non-senescence contexts such as inflammatory or immune activation responses, leading to cross-reactivity and false-positive results (Gorgoulis et al, Cell 2019; Ogrodnik et al, Cell 2024). All the above motivated the scientific community to propose a guideline algorithm for accurate senescence detection (Gorgoulis et al, Cell 2019, Kohli et al, Nat Protoc 2021). The authors should clearly show the induction of cellular senescence (and its inactivation) in their cellular settings by applying co-stainings with established senescence markers according to this algorithm. In this manner, they will prove that the senescence phenotype (and its inactivation) presented in the current work is true. In line with the above, implementation of the Fridman signature although helpful is not sufficient, as it presents some flaws related to its specificity (Ntintas et al, FEBS Bio 2025). The signature was derived from literature-based meta-analysis rather than high-throughput omics data, and many of the original reference studies relied on indirect or non-specific senescence markers, including oxidative stress genes and cell cycle regulators, whose interpretation is now considered not specific to define true senescence (Fridman and Tainsky, Oncogene 2008). In continuation to the previous, the authors state in lines 261-262 that "relapsed cells acquire a hybrid state, maintaining residual senescence features, while engaging proliferation and growth signalling programs". This enhances the above concerns whether the phenotype identified by the authors as cellular

senescence is indeed a true and full senescence program and not a transient and irrelevant state (for instance an outcome of oncogene addiction that can be related to growth arrest). Cells that escape from senescence are distinct from their paternal (senescent) ones acquiring new properties (altered genome and epigenome, increased invasion, resistance to anticancer agents and others) that have been related to cancer relapse (Milanovic et al Nature 2018; Galanos et al, NCB 2016; Zampetidis et al, Mol Cel 2021).

We are grateful to the reviewer for the thoughtful and detailed discussion of senescence markers and for highlighting recent consensus recommendations. We fully agree with the general premise that no single marker is sufficient to define cellular senescence. We agree that the proposed guideline algorithms provide important structure to the field. At the same time, as also acknowledged in these works, senescence signatures are context-dependent, varying with:

- the type of insult (e.g. OIS, therapy-induced senescence, replicative senescence, etc.), and
- the cell type and genetic background.

Our work focuses on oncogene inactivation-induced senescence (OIIS), a relatively underexplored senescence subtype. The majority of the literature (including many of the cited studies above) pertains to other senescence triggers such as classical oncogene activation (OIS) or chemotherapy-induced senescence. We do not expect a single universal marker set to apply identically across all senescence subtypes and we fully agree in accordance to the guidelines that a multi-parametric assessment is required:
We therefore combine several independent readouts:

- Cytoplasmic marker: SA-β-gal staining with characteristic enlarged, flattened morphology.
- cell-cycle state: durable cell-cycle arrest assessed by proliferation assays and cell-cycle profiles after oncogene inactivation.
- Cell-cycle regulators: p21 upregulation and p16 downregulation at mRNA and protein level, consistent with existing description in literature
- Secretory phenotype: induction of SASP factors (e.g. IL-6, MMP-3) and cytokine/ECM-remodeling signatures.

- Transcriptiome analysis

We fully acknowledge that each of these markers alone is not senescence-specific. However, taken together, we believe this multimodal combination provides sufficiently robust evidence that the observed state is indeed senescence rather than a simple transient arrest.

The authors provide some potential explanations on why the autophagy-targeted senolytic therapy did not turn out effective in vivo. This issue though remains a fundamental flaw of the current work, jeopardising its importance for developing new therapeutic avenues.

We agree with the reviewer that, as presented, our autophagy experiments might not be supportive of strong conclusions regarding the role of autophagy in OIIS escape or senolysis in our model. In light of these valid concerns, and to avoid overinterpreting our data, we have decided to remove the autophagy/senolytic part entirely from the revised manuscript. Removing this part of the experiments was also suggested by Reviewer 1. We consider this an interesting question for future, dedicated studies using more comprehensive genetic and pharmacological approaches, but no longer make claims in the present manuscript. We believe this change strengthens the manuscript by sharpening its focus on the central and best-supported aspect of our work: the characterization of OIIS and its genetic, metabolic, cytogenetic, and microenvironmental hallmarks. We now explicitly emphasize this conceptual focus in the revised Abstract and Discussion.
